# Heteroresistance to cefiderocol in carbapenem-resistant *Acinetobacter baumannii* in the CREDIBLE-CR study was not linked to clinical outcomes: a *post hoc* analysis

**DOI:** 10.1128/spectrum.02371-23

**Published:** 2023-11-15

**Authors:** Christopher Longshaw, Anne Santerre Henriksen, Dana Dressel, Michelle Malysa, Christian Silvestri, Miki Takemura, Yoshinori Yamano, Takamichi Baba, Christine M. Slover

**Affiliations:** 1 Medical Affairs, Shionogi B.V., London, United Kingdom; 2 IHMA, Schaumburg, Illinois, USA; 3 Laboratory for Drug Discovery and Disease Research, Shionogi & Co., Ltd., Osaka, Japan; 4 Biostatistics Center, Shionogi & Co., Ltd., Osaka, Japan; 5 Medical Affairs, Shionogi Inc., Florham Park, New Jersey, USA; University of California, San Diego, La Jolla, California, USA

**Keywords:** *Acinetobacter baumannii*, carbapenem resistance, cefiderocol, CREDIBLE-CR, heteroresistance, mortality, population analysis profiling

## Abstract

This study aimed to determine the proportion of heteroresistance in carbapenem-resistant *Acinetobacter calcoaceticus-baumannii* complex (CRAB) isolates in the cefiderocol arm of the randomized, Phase 3 CREDIBLE-CR study by population analysis profiling (PAP) and to determine whether there is any correlation between heteroresistance and clinical outcomes. PAP phenotypes [PAP-susceptible (PAP-S), PAP-heteroresistant (PAP-HR), or PAP-resistant (PAP-R)] were determined for baseline CRAB isolates after growing for 72 hours on agar plates containing twofold dilutions of cefiderocol (0.5–64 µg/mL). Clinical cure, microbiological eradication, and all-cause mortality (ACM) were analyzed by PAP phenotype. Only descriptive statistics were performed. Of the 38 CRAB isolates, 36 were susceptible and 2 were non-susceptible by broth microdilution (reference method), while 18 (47.4%) isolates were PAP-HR, 7 (18.4%) were PAP-S, and 13 (34.2%) were PAP-R. ACM by the end of study (end of treatment + 28 days) was 22.2% (4/18) for patients with PAP-HR isolates, 100% (7/7) with PAP-S isolates, and 61.5% (8/13) with PAP-R isolates. Among patients with PAP-HR isolates, 77.8% (14/18) had clinical cure and 38.9% (7/18) had microbiological eradication at test of cure. Among patients with PAP-S isolates, none had clinical cure or microbiological eradication. For patients with PAP-R isolates, clinical cure [23.1% (3/13)] and microbiological eradication [15.4% (2/13)] rates were low at test of cure. Using the PAP method, heteroresistance was detected in CRAB isolates in the cefiderocol arm in the CREDIBLE-CR study. However, heteroresistance was not associated with increased mortality or worse clinical and microbiological outcomes compared with patients with non-heteroresistant isolates.

**IMPORTANCE:**

The population analysis profiling (PAP) test is considered the “gold standard” method to detect heteroresistance. It exposes bacteria to increasing concentrations of antibiotics at high cell densities to detect any minority resistant subpopulations that might be missed by the low inoculums used for reference susceptibility tests. However, its clinical relevance has not been well established. In the CREDIBLE-CR study, a numerically increased all-cause mortality was observed in the cefiderocol arm relative to the best available therapy arm for patients with *Acinetobacter* spp. infections. Heteroresistance has independently been proposed by another research group as a potential explanation of the mortality difference. An analysis of the baseline carbapenem-resistant *Acinetobacter calcoaceticus-baumannii* complex isolates from patients treated with cefiderocol in the CREDIBLE-CR study showed the highest clinical cure rate and the lowest mortality for patients with PAP-heteroresistant isolates compared with PAP-susceptible or PAP-resistant isolates. These findings contradict the abovementioned hypothesis that heteroresistance contributed to the increased mortality.

## INTRODUCTION

Members of the *Acinetobacter calcoaceticus-baumannii* complex are opportunistic pathogens that have developed multiple resistance mechanisms to a variety of antibiotics, including carbapenems (1, 2). Carbapenem-resistant (CR) *A. baumannii* (CRAB) is considered by the World Health Organization to be one of the most challenging pathogens in healthcare settings, including intensive care units (ICUs) (3). Because morbidity and mortality rates remain high (39%–57%) and treatment options are limited, new antibiotics are urgently needed (3 – 6).

In the randomized, pathogen-focused, descriptive Phase 3 CREDIBLE-CR study, a numerically increased mortality was observed at all study visits in the cefiderocol arm relative to the best available therapy (BAT) arm in patients infected with CRAB, mainly with nosocomial pneumonia (NP) or bloodstream infection (BSI)/sepsis (7). Although the mortality rate in the cefiderocol arm was found to be similar to that observed in previous randomized, controlled clinical studies of CRAB infections (5, 6), the difference in mortality was partially explained by imbalances in some baseline prognostic risk factors between treatment arms at randomization, including fewer patients with prior or ongoing shock in the BAT arm (6, 7).

Heteroresistance is a phenomenon based on *in vitro* observations under antibiotic pressure whereby a small subpopulation of cells seemingly resistant to an antibiotic may be recovered from cultures of otherwise susceptible bacteria. As the heteroresistant cells that grow under selective antibiotic pressure are unstable, they revert to a susceptible phenotype when the antibiotic pressure is removed (8, 9). This phenomenon remains poorly understood in terms of underlying genetic mechanisms and its clinical relevance (10 – 12). Heteroresistance was first described in methicillin-resistant *Staphylococcus aureus* initially for methicillin (13) but later for vancomycin, which was then characterized as heterogeneous vancomycin-intermediate *S. aureus* (hVISA) (12), and daptomycin (14). More recently, heteroresistance has been used to describe similar growth phenotypes in *A. baumannii* and other Gram-negative bacteria exposed to a range of antibiotics from different classes, such as colistin, tigecycline, ampicillin-sulbactam, amikacin, and carbapenems (8, 15, 16) and even to fluconazole in *Candida glabrata* (17). Heteroresistance to cefiderocol has also been recently reported in CRAB (18, 19), *Klebsiella pneumoniae* (20, 21), and *Pseudomonas aeruginosa* (22).

As the increased all-cause mortality (ACM) in the CREDIBLE-CR study was observed in polymicrobial infections involving *A. baumannii*, it has been suggested that heteroresistance in CRAB might have contributed to the numerical ACM difference between cefiderocol-treated patients and patients receiving BAT (18, 23).

In the current study, we evaluated CRAB isolates from the cefiderocol arm of the CREDIBLE-CR study by population analysis profiling (PAP) to measure the proportion of heteroresistance to cefiderocol in these clinical isolates and to determine whether there might be any relationship between heteroresistance and mortality or other clinical and microbiological outcomes.

## RESULTS

### PAP susceptibility phenotypes of 38 CRAB isolates

**Fig 1:**
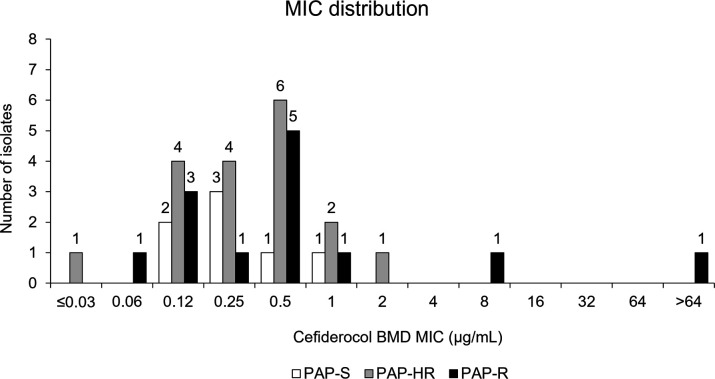
Distribution of cefiderocol minimum inhibitory concentration by broth microdilution for carbapenem-resistant *Acinetobacter calcoaceticus-baumannii* complex isolates in the cefiderocol arm in the CREDIBLE-CR study by population analysis profiling phenotype.

A total of 39 patients in the cefiderocol arm of the CREDIBLE-CR study had CRAB identified as a baseline pathogen, of which 38 were available for further evaluation (36 CR *A. baumannii* and 2 CR *Acinetobacter nosocomialis*). Detailed microbiology results were not available for one isolate. Although 36 of 38 isolates were defined as susceptible to cefiderocol by the reference broth microdilution (BMD) method according to Clinical and Laboratory Standards Institute (CLSI) breakpoints (MIC range ≤0.03–2 µg/mL), only 7 of these (19.4%) BMD-susceptible isolates were categorized as susceptible by the PAP method (PAP-S); 18 isolates (50.0%) were defined as heteroresistant (PAP-HR), and 11 (30.6%) were defined as resistant (PAP-R) (Table 1). Two isolates that were categorized as non-susceptible by BMD [one intermediate (MIC = 8 µg/mL) and one resistant (MIC > 64 µg/mL)] were both defined as resistant by PAP (Table 1). PAP results for each isolate are shown in Fig. S1.1 to S1.38. Cefiderocol susceptibility determined by disk diffusion was in agreement with BMD MIC values, except for the BMD-intermediate isolate, which was categorized as PAP-R and showed a diameter of 16 mm (susceptible), and one BMD-susceptible isolate, categorized as PAP-S, which had a diameter of 9 mm (non-susceptible) (Table 1). Disk diffusion test inhibition zone diameters ranged between 18 and 27 mm for PAP-S, 16 and 23 mm for PAP-HR, and 6 and 26 mm for PAP-R isolates. Cefiderocol MIC distribution by BMD for PAP-S, PAP-HR, and PAP-R isolates is shown in Fig. 1.

**TABLE 1:** Cefiderocol susceptibility by broth microdilution and disk diffusion and by population analysis profiling of 38 baseline carbapenem-resistant *Acinetobacter calcoaceticus-baumannii* complex isolates in the cefiderocol arm in the CREDIBLE-CR study^*a*^

BMD phenotype	Disk diffusion phenotype		PAP phenotype at 72-hour time point		
	S (inhibition zone ≥ 15 mm) *n* (row %)	Non-S (inhibition zone < 15 mm) *n* (row %)	S *n* (row %)	HR *n* (row %)	R *n* (row %)
S (MIC ≤ 4 µg/mL; *n* = 36)	35 (97.2)	1 (2.8)	7 (19.4)	18 (50.0)	11 (30.6)
I (MIC = 8 µg/mL; *n* = 1)	1 (100)	0	0	0	1 (100)
R (MIC ≥ 16 µg/mL; *n* = 1)	0	1 (100)	0	0	1 (100)
Total (*N* = 38)	36 (94.7)	2 (5.3)	7 (18.4)	18 (47.4)	13 (34.2)

^*a*^ *n*, number of isolates in the category.

### Characteristics of patients with CRAB infections by PAP susceptibility phenotype

We analyzed baseline demographics and clinical characteristics for 38 patients with CRAB infections in the cefiderocol arm (Table 2). The median age was 67.5 years (range: 23–91) and was similar across subsets of patients with PAP-S, PAP-HR, and PAP-R isolates. Overall, most patients [71.1% (27/38)] had NP, followed by BSI/sepsis in 26.3% of patients (10/38), and 2.6% (1/38) had complicated urinary tract infection (cUTI).

**TABLE 2:** Baseline demographics and clinical characteristics of 38 patients with carbapenem-resistant *Acinetobacter calcoaceticus-baumannii* complex isolates overall and by population analysis profiling phenotype in the carbapenem-resistant microbiological intent-to-treat population in the cefiderocol arm in the CREDIBLE-CR study^*a*^

	Overall (*N* = 38)	PAP-S (*N* = 7)	PAP-HR (*N* = 18)	PAP-R (*N* = 13)
Age (years), median (range)	67.5 (23–91)	64 (24–78)	67.5 (23–86)	70 (24–91)
Sex (male), *n* (%)	24 (63.2)	3 (42.9)	12 (66.7)	8 (61.5)
Region, *n* (%)				
America	6 (15.8)	0 (0)	3 (16.7)	3 (23.1)
Europe	17 (44.7)	5 (71.4)	8 (44.4)	4 (30.8)
Asia	15 (39.5)	2 (28.6)	7 (38.9)	6 (46.2)
Clinical diagnosis, *n* (%)				
VAP/HAP/HCAP	27 (71.1)	4 (57.1)	12 (66.7)	11 (84.6)
BSI/sepsis	10 (26.3)	3 (42.9)	6 (33.3)	1 (7.7)
cUTI	1 (2.6)	0 (0)	0 (0)	1 (7.7)
APACHE II score, median (range)	16.5 (5–29)	18 (12–24)	17 (7–29)	14.5 (5–27)
SOFA score, median (range)	6 (1–17)	6 (3–9)	6.5 (1–17)	6 (1–14)
Previous treatment failure, *n* (%)	23 (60.5)	4 (57.1)	12 (66.7)	9 (69.2)
ICU, *n* (%)	32 (84.2)	7 (100)	16 (88.9)	9 (69.2)
Septic shock within 31 days prior to randomization or at screening, *n* (%)	9 (23.7)	1 (14.3)	2 (11.1)	6 (46.2)
Charlson Comorbidity Index score	5 (0–11)	6 (3–10)	4 (0–11)	6 (0–10)
Creatinine clearance grade, *n* (%)				
ARC	7 (18.4)	2 (28.6)	5 (27.8)	0 (0)
Normal	9 (23.7)	1 (14.3)	6 (33.3)	2 (15.4)
Mild	10 (26.3)	1 (14.3)	3 (16.7)	6 (46.2)
Moderate	7 (18.4)	2 (28.6)	3 (16.7)	2 (15.4)
Severe	5 (13.2)	1 (14.3)	1 (5.6)	3 (23.1)
Plasma *C*_min_, range (µg/mL)	4.28–59.4	14.4–29.9	4.28–47.7	5.5–59.4
Plasma *C*_min_/MIC	0.4–573.3 [*n* = 27]	26.4–249.2 [*n* = 4]	3.3–573.3 [*n* = 15]	0.4–313.3 [*n* = 8]
Day of death, range	3–45	6–45	4–27	3–19
Treatment duration, range (days)	2–22	2–19	4–22	3–22
Combination treatment, *n* (%)	8 (21.1)	3 (42.9)	3 (16.7)	2 (15.4)
Polymicrobial infection, *n* (%)	12 (31.6)	2 (28.6)	5 (27.8)	5 (38.5)

^*a*^ APACHE II, Acute Physiology And Chronic Health Evaluation II; ARC, augmented renal clearance; *C*_min_, minimum plasma concentration; HAP, hospital-acquired pneumonia; HCAP, healthcare-associated pneumonia; SOFA, Sequential Organ Failure Assessment; and VAP, ventilator-associated pneumonia.

The median Acute Physiology And Chronic Health Evaluation II (APACHE II) score was 16.5 (range: 5–29) and was slightly lower for the subset of patients with PAP-R CRAB isolates. The median Sequential Organ Failure Assessment (SOFA) score was 6 (range: 1–17) and was similar across the subsets of patients. Nearly all patients (84.2%) were in the ICU, including all patients (100%) with PAP-S isolates, 88.9% with PAP-HR isolates, and 69.2% with PAP-R isolates. Nearly half [46.2% (6/13)] of the patients with PAP-R isolates had prior or ongoing septic shock at randomization, compared with two (11.1%) and one (14.3%) among those with PAP-HR and PAP-S isolates, respectively. Charlson Comorbidity Index (CCI) was numerically lower for patients with PAP-HR isolates than for patients with PAP-S and PAP-R isolates (Table 2).

Treatment duration with cefiderocol ranged between 2 and 22 days and was similar across the different subsets of patients. Combination therapy was given to eight patients (21.1%) overall (Table 2).

### Comparison of ACM by PAP susceptibility phenotype

**Fig 2:**
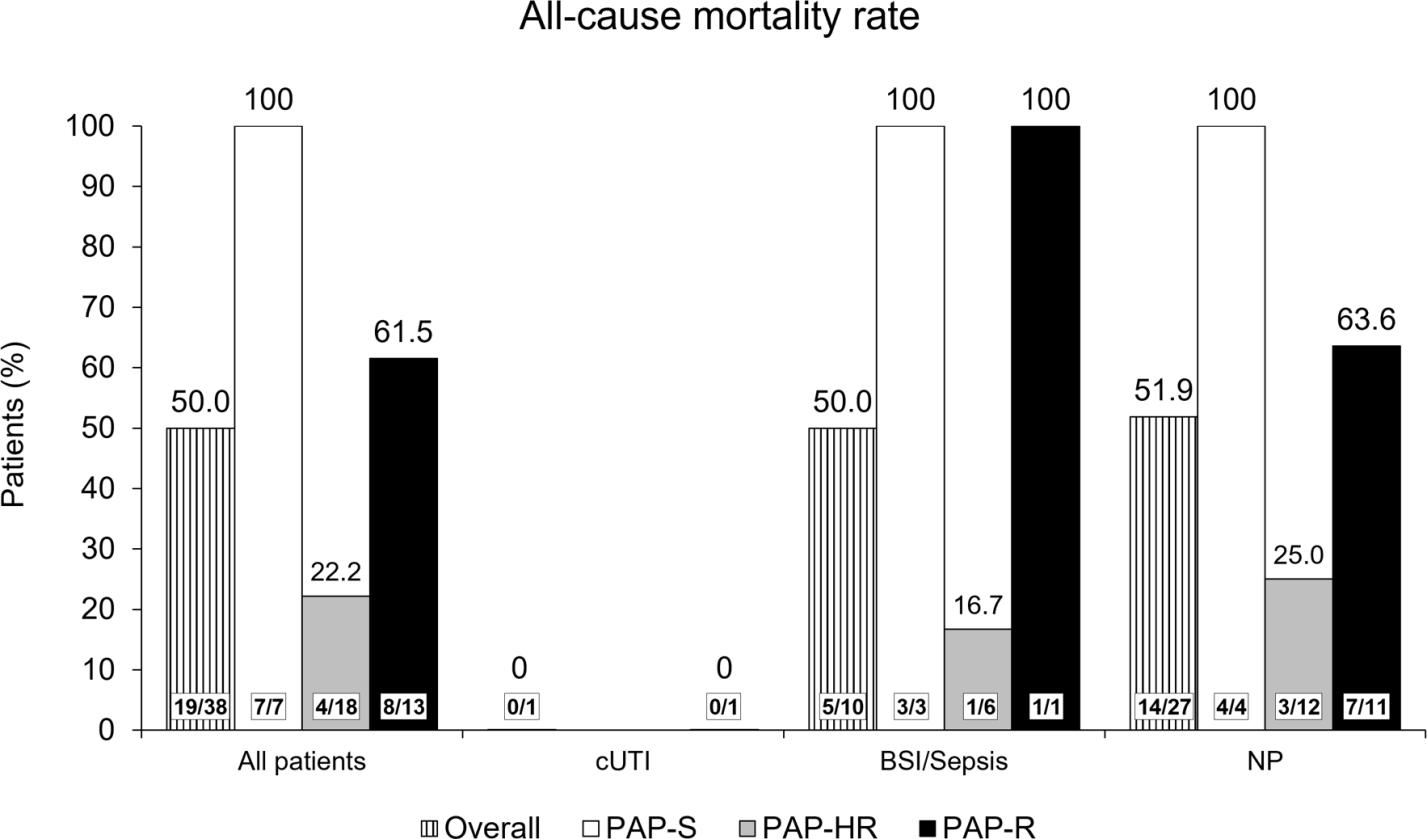
All-cause mortality rates at the end of study overall and by clinical diagnosis and population analysis profiling phenotype in the cefiderocol arm in the CREDIBLE-CR study.

The overall mortality rate by the end of study (EOS) among the 38 patients with CRAB infections was 50.0% (19/38). No correlation was found between the PAP phenotype and ACM; in fact, the lowest rate of mortality was found in the subset of 18 patients with CRAB infections categorized as PAP-HR [22.2% (4/18)]. All seven patients with PAP-S CRAB isolates died, as did 8/13 patients (61.5%) with PAP-R CRAB isolates (Fig. 2).

By clinical diagnosis, one patient had cUTI with a PAP-R CRAB isolate and the patient survived to EOS. Among 10 patients with BSI/sepsis, five patients (50.0%) died by EOS, of whom three had PAP-S isolates, one had PAP-R, and one had PAP-HR (Fig. 2). Among 27 patients with NP, 3 of 12 patients with PAP-HR isolates (25.0%) died, as did all 4 patients (100%) with PAP-S isolates, and 7 of 11 patients (63.6%) with PAP-R isolates (Fig. 2). Mortality rates were similar for patients with ventilator-associated pneumonia (VAP) and hospital-acquired pneumonia (HAP)/ healthcare-associated pneumonia (HCAP).

By Day 14, 11 patients had died: two (11.1%) of those with PAP-HR isolates, three (42.9%) of those with PAP-S isolates, and six (46.2%) of those with PAP-R isolates. By Day 28 and EOS, lower ACM was seen in patients with PAP-HR isolates than in patients with PAP-S and PAP-R isolates (Table 3). Among patients with BSI/sepsis, all three patients with PAP-S isolates had died by Day 14, while among patients with NP, all four patients with PAP-S isolates had died by EOS (Table 3). Among patients with NP, the ACM rate at each visit was lower for patients with PAP-HR isolates than for patients with PAP-R isolates (Table 3).

**TABLE 3:** All-cause mortality at Day 14, Day 28, and the end of study in 38 patients with carbapenem-resistant *Acinetobacter calcoaceticus-baumannii* complex infections overall and by clinical diagnosis and population analysis profiling phenotype in the cefiderocol arm in the CREDIBLE-CR study

Diagnosis visit^*b*^	All-cause mortality *n* (% of total number of patients)^*a*^			
Overall	Overall (*N* = 38)	PAP-S (*N* = 7)	PAP-HR (*N* = 18)	PAP-R (*N* = 13)
Day 14	11 (28.9)	3 (42.9)	2 (11.1)	6 (46.2)
Day 28	15 (39.5)	3 (42.9)	4 (22.2)	8 (61.5)
EOS	19 (50.0)	7 (100)	4 (22.2)	8 (61.5)
cUTI	Overall (*N* = 1)	PAP-S (*N* = 0)	PAP-HR (*N* = 0)	PAP-R (*N* = 1)
Day 14	0 (0)	–^*c*^	–	0 (0)
Day 28	0 (0)	–	–	0 (0)
EOS	0 (0)	–	–	0 (0)
BSI/sepsis	Overall (*N* = 10)	PAP-S (*N* = 3)	PAP-HR (*N* = 6)	PAP-R (*N* = 1)
Day 14	3 (30.0)	3 (100)	0 (0)	0 (0)
Day 28	5 (50.0)	3 (100)	1 (16.7)	1 (100)
EOS	5 (50.0)	3 (100)	1 (16.7)	1 (100)
VAP/HAP/HCAP	Overall (*N* = 27)	PAP-S (*N* = 4)	PAP-HR (*N* = 12)	PAP-R (*N* = 11)
Day 14	8 (29.6)	0 (0)	2 (16.7)	6 (54.5)
Day 28	10 (37.0)	0 (0)	3 (25.0)	7 (63.6)
EOS	14 (51.9)	4 (100)	3 (25.0)	7 (63.6)

^*a*^ *n*, number of patients in the category; *N*, total number of patients in the category.

^*b*^ EOS, end of study [end of treatment + 28 days (±3) days].

^*c*^ –, not applicable.

The median CCI was 7 (range: 3–11) for patients who died (*n* = 19) and 4 (range: 0–7) for patients who survived (*n* = 19). The median APACHE II score and median SOFA score were also higher for patients who died than for patients who survived by EOS. Additionally, eight of nine patients with septic shock prior to or at randomization died (Table S1).

All-cause mortality rates by EOS did not correlate with BMD cefiderocol MIC values or by PAP susceptibility phenotype (Table 4).

**TABLE 4:** All-cause mortality by the end of study by population analysis profiling phenotype and cefiderocol minimum inhibitory concentration values by broth microdilution in patients with carbapenem-resistant *Acinetobacter calcoaceticus-baumannii* complex infections in the cefiderocol arm in the CREDIBLE-CR study

	All-cause mortality, % (*n*/*N*’)^*a*^		
**BMD MIC (µg/mL)**	**PAP-S**	**PAP-HR**	**PAP-R**
Overall	100 (7/7)	22.2 (4/18)	61.5 (8/13)
≤0.03	0	0 (0/1)	0
0.06	0	0	0 (0/1)
0.12	100 (2/2)	25.0 (1/4)	33.3 (1/3)
0.25	100 (3/3)	0 (0/4)	0 (0/1)
0.5	100 (1/1)	50.0 (3/6)	80.0 (4/5)
1	100 (1/1)	0 (0/2)	100 (1/1)
2	0	0 (0/1)	0
4	0	0	0
8	0	0	100 (1/1)
16	0	0	0
32	0	0	0
64	0	0	100 (1/1)
>64	0	0	0

^*a*^ *n*, number of patients in the category; *N*’, total number of patients in the category.

### Comparison of clinical cure and microbiological eradication by PAP susceptibility phenotype

**Fig 3:**
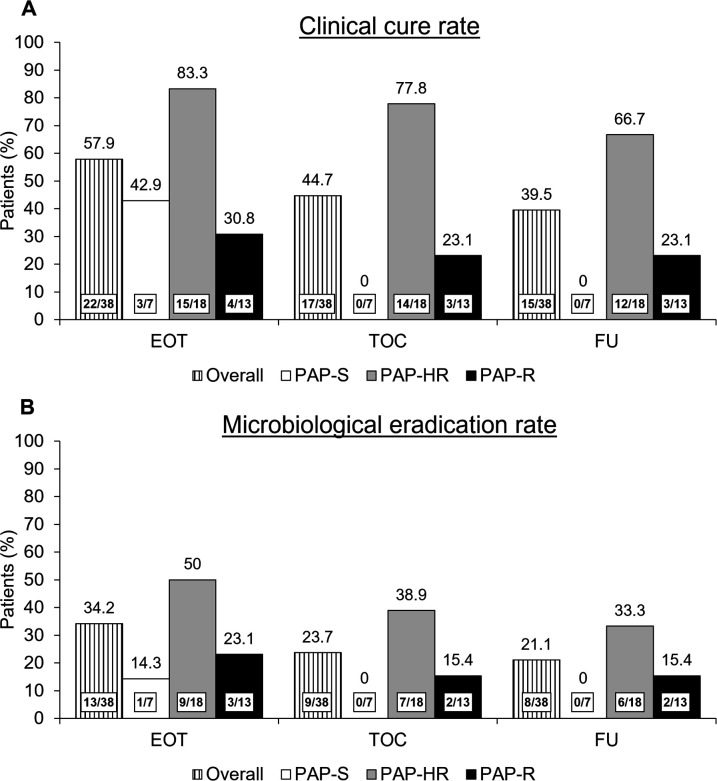
Clinical cure (**A**) and microbiological eradication (**B**) according to susceptibility phenotype by population analysis profiling among 38 patients with carbapenem-resistant *Acinetobacter calcoaceticus-baumannii* complex infections in the cefiderocol arm in the CREDIBLE-CR study. FU, follow-up.

At the end of treatment (EOT) visit, 83.3% of patients with PAP-HR infections were judged to have clinical cure compared with 42.9% of patients with PAP-S isolates and 30.8% of patients with PAP-R isolates (Fig. 3A). By the test of cure (TOC) visit, 77.8% of patients with PAP-HR infections and 23.1% of patients with PAP-R isolates achieved clinical cure, while none of the patients with PAP-S infection met this endpoint (Fig. 3A).

Microbiological eradication rates were seen to be highest at each study visit for infections with PAP-HR CRAB isolates compared with PAP-S isolates and PAP-R isolates (Fig. 3B). Microbiological persistence rates did not correlate with any PAP susceptibility phenotype (data not shown). Among the 38 CRAB isolates, an on-therapy fourfold MIC increase was previously confirmed for three isogenic isolates in three patients, of which two isolates were PAP-S, one was PAP-R, and none was PAP-HR.

### Distribution of beta-lactamase enzymes by PAP susceptibility phenotype

The most frequent beta-lactamase enzyme detected was oxacillinase-23 (OXA-23), which was present in 31 of 38 CRAB isolates, 9 of which also carried the Temoneira Class A beta-lactamase (TEM-1) (Table 5). New Delhi metallo-beta-lactamase 1 (NDM-1) enzyme was found in two isolates. No *Pseudomonas* extended resistance (PER) enzyme was detected. PAP phenotype was not associated with the presence of any specific beta-lactamase.

**TABLE 5:** Distribution of beta-lactamases in 38 carbapenem-resistant *Acinetobacter calcoaceticus-baumannii* complex isolates by population analysis profiling phenotype in the cefiderocol arm in the CREDIBLE-CR study^*a*^

Beta-lactamase enzyme^*b*^	Overall (*n*)	PAP-S (*n*)	PAP-HR (*n*)	PAP-R (*n)*
OXA-23	20	4	11	5
OXA-24	2	0	1	1
OXA-72	2	1	0	1
TEM-1, OXA-23	9	2	3	4
TEM-1, OXA-24	1	0	1	0
TEM-1, OXA-72	0	0	0	0
TEM-1, NDM-1	1	0	0	1
TEM-OSBL; OXA-23	1	0	1	0
TEM-1; NDM-1; OXA-23	1	0	1	0
No acquired beta-lactamase detected	1	0	0	1
Total	38	7	18	13

^*a*^ *n*, number of isolates with beta-lactamase enzymes.

^*b*^ OSBL, original-spectrum beta-lactamase; OXA, oxacillinase.

## DISCUSSION

In this study of CRAB isolates from patients in the cefiderocol arm of the CREDIBLE-CR study, 47% of isolates met the definition of heteroresistance using population analysis profiling or PAP, which is generally considered to be the gold standard methodology. This percentage was similar to that reported previously by Choby et al. (i.e., 59% of 108 CRAB isolates collected from a surveillance study in Georgia, USA) (18). In that letter, the authors speculated that heteroresistance could be a plausible explanation for the increased mortality observed in patients with CRAB infections in the cefiderocol arm of the CREDIBLE-CR study (7, 18, 23). However, the current study has found no correlation between infections caused by isolates that are defined as heteroresistant to cefiderocol by PAP and clinical outcomes, including ACM and clinical cure, in the cefiderocol arm of the CREDIBLE-CR study. Furthermore, while it might be expected that isolates with heteroresistant subpopulations could have an increased risk of microbiological failure or treatment-emergent resistance, neither bacterial persistence nor increases in MIC from baseline during therapy were associated with the heteroresistant phenotype. Additionally, no correlation was found between zone diameter, the presence of microcolonies or double zones around the disks (data not shown), and the PAP phenotype.

Interestingly, 30.6% of isolates that were categorized as susceptible by the CLSI reference BMD or disk diffusion methods met the definition for resistance by the PAP method, with viable counts >50% of the no-drug control observed on plates containing 16, 32, or 64 µg/mL of cefiderocol. This discordance in the phenotypes merits a more detailed investigation to better understand the biological explanation for the incongruence between methods; however, a closer analysis of these BMD-susceptible/PAP-R isolates also found no significant correlation with clinical outcomes.

There are a number of possible considerations that might explain the different growth phenotypes observed by population analysis profiling. For this study, PAP was performed in line with the method recommended by Shermann et al. for detecting heteroresistance to colistin in *A. baumannii* (9). According to this method, the growth of isolates in the presence of antibiotics was read at 24, 48, and 72-hour endpoints, with the final interpretation after 72 hours to allow adequate time for slow-growing subpopulations to grow sufficiently to be counted (24); despite this, there was no consistency in the growth pattern of isolates over time, and no link between mortality and PAP phenotypes was observed at earlier time points (data not shown). The baseline CRAB isolates were not exposed previously to cefiderocol because patients with prior cefiderocol treatment were excluded from the CREDIBLE-CR study. Thus, any phenotypic changes in these isolates could be considered as *de novo* phenotypic changes in susceptibility under the culture conditions due to the selection of low-frequency, phenotypically resistant subpopulations or induction of an antibiotic-tolerant phenotype. It has been suggested in a previous work, which tested 41 clinical isolates of Gram-negative species against 28 different antibiotics, that *Acinetobacter* spp. may adapt rapidly to grow in Mueller–Hinton broth by displaying 18%–20% higher fitness (15). It has been shown that variability in culture conditions can have a direct impact on the growth and *in vitro* susceptibility of Gram-negative bacteria (25). It was also suggested that heterogeneity in bacterial populations, including persisters, susceptible, and heteroresistant cells, may confer a fitness advantage for the whole population, allowing it to maintain colonization in patients (26).

Stracquadanio et al. showed that CRAB isolates that were PAP-HR to cefiderocol were unstable, and after two serial passages in the absence of cefiderocol, the isolates were susceptible with reduced MIC values (19). Because heteroresistant isolates growing under antibiotic pressure are not stable, detection of heteroresistant isolates in hospital laboratories remains challenging, as the methodology is time-consuming and labor-intensive (8, 15). Guiding antibiotic selection for treatment, therefore, is mainly reliant on standard susceptibility testing with BMD or disk diffusion.

The mechanisms behind the phenotypic heteroresistance are unclear. However, spontaneous tandem amplification of sequences, point mutations or small deletions (15), and upregulation of efflux pumps (16) were described in PAP-HR Gram-negative pathogens. Many clinical isolates of CRAB are known to carry mobile genetic elements such as IS*Aba*1, which may contribute to the development of resistance during selection pressure through promoting gene amplification or insertion into or upstream of resistance determinants, leading to up- or downregulation of gene expression (e.g., OXA-23) (27). These genetic changes may contribute to heterogeneous growth phenotypes in the presence of beta-lactams at high concentrations.

Population analysis profiling has been described as the gold standard method for detecting heteroresistance *in vitro*. The methodology was originally described for MRSA and developed to detect hVISA isolates growing in the presence of vancomycin, but there was a debate about its sensitivity, specificity, and clinical relevance (12, 28). The original PAP method was later replaced by the modified PAP-area under the concentration-time curve (AUC) ratio method, which compared the ratio of AUC for the test isolate with a reference hVISA Mu3 isolate; however, this method provided low sensitivity despite high specificity (29). The application of the PAP or modified PAP methodology to species other than hVISA is not unequivocally justified in the absence of stable heteroresistant reference strains for comparison. Other features of the PAP methodology may also make it unreliable for pathogen/drug combinations other than hVISA/glycopeptides, and their contribution to any observed phenotypes should be carefully considered and minimized before the relevance of *in vitro* phenotypes can be translated into clinical outcomes.

Established reference methods for antimicrobial susceptibility testing such as broth and agar dilution as well as Kirby-Bauer disk diffusion are highly standardized and require inoculation at relatively low cell density (~5 × 10^5^ CFU/mL). However, in order to detect small (rare) heteroresistant subpopulations, which may represent only one in 1 × 10^6^ of the total cell population, the PAP method uses a much higher cell-density inoculum from 10^8^ to 10^9^ CFU/mL (1,000–10,000-fold higher density) compared to the reference method. Even in the *in vitro* BMD assay, increasing the inoculum of bacteria often results in a shift to higher MIC values, in part because the same concentration of an antibiotic is no longer sufficient to inhibit the growth of a higher number of bacterial cells. Interestingly, the inoculum effect has been shown to differ with different modes of action and is most pronounced for beta-lactams, including cefiderocol, and perhaps reflects the time-dependent pharmacodynamic driver (%fT_>MIC_) of this class of antibiotics (30 – 34). Care should be taken not to misinterpret the inoculum effect as reduced phenotypic susceptibility and any increase in MIC relative to an increased inoculum needs to be taken into account in the PAP method, especially when the definitions of susceptible, resistant, and heteroresistant are based on clinical interpretative breakpoints set relative to the low cell density inoculum.

The only reference method recommended by CLSI, the US Food and Drug Administration, and the European Committee on Antimicrobial Susceptibility Testing (EUCAST) for testing cefiderocol susceptibility is the broth microdilution method using iron-depleted, cation-adjusted Mueller–Hinton broth (ID-CAMHB). The agar dilution method is not recommended and susceptibility breakpoints by agar dilution have not been established. This is mainly due to the observations from a study by Albano et al. comparing MICs for cefiderocol between broth microdilution and agar dilution methods, which showed poor essential agreement as well as poor categorical agreement, with high rates of major errors and minor errors especially for *A. baumannii* (35). Most importantly, a proportion of isolates with low MIC values by BMD displayed a much wider range of MIC values by agar dilution (e.g., up to 32–64-fold increased MICs) (35)—this is despite agar dilution using the same starting inoculum as BMD. Therefore, survival of populations apparently at higher MICs in the PAP method may not only be due to the inoculum effect but may also be in part explained by differences in MIC by agar dilution compared to BMD methods.

Beta-lactamase production might also be a complicating factor. A recent study of carbapenemase-producing *Escherichia coli* isolates suggested that the release of carbapenemases from dying cells under the PAP culture conditions led to hydrolysis of meropenem, which allowed the growth of *E. coli* cells and mimicked heteroresistance to meropenem (10). The authors suggested that the role of released beta-lactamases from dying cells hydrolyzing beta-lactam antibiotics in culture cannot be excluded, and the results of the PAP test should be cautiously implemented in the management of patients with multidrug-resistant Gram-negative bacterial infections (10). The role of beta-lactamases was previously linked to the paradoxical growth effect of beta-lactam antibiotics by Ikeda and Nishino (36). In a recent case report on NDM-5-producing *K. pneumoniae* infection, the isolate was heteroresistant to cefiderocol with a PAP test MIC value of ≥32 µg/mL and susceptible with 18 mm zone diameter on disk diffusion test according to both EUCAST and CLSI interpretive criteria (20, 37, 38).

Thus, the higher cell density of the starting inoculum level, the use of agar dilution plates, and the longer incubation time at 37°C, which may increase the rate of cefiderocol degradation both naturally and due to the release of exogenous beta-lactamases into the agar matrix, are all likely to introduce a bias in the PAP test toward survival at higher cefiderocol concentrations (33, 35).

It has been postulated that the detection of heteroresistance using the PAP method could be linked to treatment failure and/or mortality. However, two small studies have failed to prove the association between heteroresistance in *A. baumannii* and treatment failure, and between heteroresistance in *P. aeruginosa* and mortality (39, 40). As a result of the poor predictive power of the PAP methodology, routine screening for heteroresistance by clinical microbiology laboratories is not recommended (24).

We have found in the current analysis that patient characteristics varied across the subsets of the CREDIBLE-CR patients in terms of prognostic factors, such as ICU admission, APACHE II score, prior or ongoing shock, and CCI, which likely contributed to overall patient all-cause mortality. Among patients who died in the current study, we found higher baseline APACHE II score, SOFA score, CCI, a higher proportion of patients with moderate-severe renal impairment, septic shock, and prior organ failure compared with patients who survived (Table S1). However, these findings should be interpreted carefully due to the small patient numbers and the descriptive nature of the data. Among the patients enrolled in the prospective, randomized AIDA clinical trial, the fitness of CRAB (expressed by increased CFU/mL), as well as clinical prognostic factors (i.e., age, SOFA score, and CCI), was found to be predictors of clinical failure and mortality, suggesting that bacterial fitness at the time of randomization may be used as a stratification factor to investigate the efficacy of new antimicrobial agents against CRAB (41).

Our results showed a high frequency of heteroresistance in a global collection of CRAB isolates from the cefiderocol arm of the randomized CREDIBLE-CR study. However, heteroresistance was not linked to mortality, as patients with PAP-HR CRAB isolates had a lower ACM rate (i.e., 22.2%) than patients with PAP-S (i.e., 100%) and PAP-R (i.e., 61.5%) CRAB isolates. Furthermore, ACM at Days 14 and 28 was found to be the lowest for patients with PAP-HR isolates, and clinical cure and microbiological eradication rates were highest in the subset of patients with PAP-HR CRAB infections who had challenging infections such as BSI/sepsis and NP. The cause of death within 14 days in a number of patients with CRAB infections was linked to the deterioration of underlying comorbidities or ongoing sepsis/septic shock that was present at randomization (7).

The available pharmacokinetic data (i.e., minimum unbound plasma concentration value at Days 3 and 4) suggest that the percentage of time that the free drug concentration was greater than the MIC (*T*_>MIC_) of the isolates was 100% in nearly all patients, with the exception of patients with BMD cefiderocol-non-susceptible CRAB isolates. A previous study (42) suggested that microbiological failure may be predicted by the pharmacodynamic parameter of minimum plasma concentration (*C*_min_)/MIC ratio (i.e., ≤4). In our study, the *C*_min_/MIC ratio remained >4 for patients with cefiderocol-susceptible CRAB isolates, ruling out the possibility that patients had inadequate antibiotic plasma exposures. Additionally, population pharmacokinetic modeling suggested that the probability of target attainment for 100%*T*_>MIC_ was >90% for nearly all patients in the study in all infection sites and renal function groups, except for patients with BSI/sepsis and normal renal function (i.e., 85% probability) (43).

The role of non-PAP growth phenomena should also be considered in interpreting the data. A few atypical growth phenotypes have been reported for CRAB isolates exposed to cefiderocol, including trailing and paradoxical growth/“Eagle effect” (broth) and microcolonies (disk), which are well known to complicate the endpoint reading of cefiderocol susceptibility tests. These growth phenotypes are not unique to cefiderocol and have also been reported for other antibiotics, including a siderophore conjugate (36, 37, 44 – 46).

The limitations of this study include the small patient numbers in the three PAP categories. The growth patterns for these CRAB isolates were not consistent over time (Fig. S1); therefore, rates of clinical cure, microbiological eradication, and ACM based on interpretation of the PAP phenotype at an earlier time point could be nominally different. However, there was a tendency toward a lower ACM for isolates categorized as PAP-HR at both 24 and 48 hours than for isolates categorized as PAP-S or PAP-R at these time points. For the PAP-HR isolates, post-growth MIC values at 72 hours were not determined in comparison with their baseline MICs, and subsequent serial passages without cefiderocol were not performed to retest their susceptibility. As no detailed molecular characterization or whole-genome sequencing was performed, the presence of point mutations in genes related to iron transport or cell wall synthesis cannot be ruled out.

### Conclusions

In conclusion, we found no link between PAP heteroresistant phenotype and mortality or clinical and microbiological outcomes in cefiderocol-treated patients with CRAB infections. As previously discussed (47), we believe that the PAP methodology itself likely biases toward survival of CRAB isolates at cefiderocol concentrations that would be inhibitory by the reference standard methods used to set clinical breakpoints, and the relevance and impact of these breakpoints on the interpretation of heteroresistance need to be carefully considered. The data shown in this study do not support the previously raised hypothesis that heteroresistance was associated with the numerically increased mortality rate in the cefiderocol arm in the randomized, global, multicenter CREDIBLE-CR study. Improved *in vitro* methods need to be developed to investigate the clinical relevance of heterogeneous growth phenotypes to antibiotics in CRAB and other pathogens.

## MATERIALS AND METHODS

### Study design of the CREDIBLE-CR study

CREDIBLE-CR (NCT02714595) was an open-label, randomized, multicenter, pathogen-focused, descriptive Phase 3 clinical study to investigate the efficacy and safety of cefiderocol 2 g, 3 hour infusion, every 8 hours, or renally adjusted doses, or BAT according to local practice in patients with serious CR Gram-negative bacterial infections (7). Patients with cUTI, BSI/sepsis, VAP, HAP, and HCAP with evidence of carbapenem resistance were enrolled. Patients who had received prior treatment with cefiderocol were excluded from the study. Randomization and blinding, inclusion and exclusion criteria, treatment arms, procedures and definitions, primary and secondary endpoints, and statistical analyses have been described previously (7). Overall data collected on clinical outcomes, microbiological outcomes, vital status, baseline demographics, clinical characteristics, and susceptibility of baseline pathogens were described, analyzed, and published previously (7).

### Isolates

In the CREDIBLE-CR study, CRAB isolates were confirmed at the central laboratory (IHMA, Schaumburg, IL, USA) from appropriate biospecimens collected at randomization (baseline isolates). The species identification, susceptibility testing, on-therapy MIC increase, and detection of beta-lactamases are described elsewhere (7, 48). Carbapenem susceptibility status for Gram-negative pathogens was determined according to CLSI methods and interpreted according to CLSI breakpoints (7).

### Antibiotic susceptibility testing

The CRAB isolates were stored in glycerol stocks at –80°C in tryptic soy broth containing 15% glycerol. After recovery from storage, all isolates were retested to confirm susceptibility to cefiderocol using frozen BMD panels and disk diffusion methods following the CLSI guidelines (49, 50). Quality control strains of *E. coli* ATCC 25922 and *P. aeruginosa* ATCC 25783 were tested concurrently for both methods.

Inoculum suspensions were prepared in saline using the direct colony method at 0.5 McFarland (~1.5 × 10^8^ CFU/mL). A single inoculum for each isolate containing approximately 5 × 10^4^ CFU/well was used to perform BMD to determine MICs. MIC testing for cefiderocol was performed in ID-CAMHB. Disk diffusion testing was performed using cefiderocol 30 µg disks (Hardy, Santa Maria, CA, USA) on Mueller–Hinton agar (MHA) plates (Remel, Thermo Scientific, Lenexa, KS, USA) streaked with 0.5 McFarland cell suspension. BMD and agar plates were incubated for 20–24 hours at 36°C ± 1°C in a non-CO_2_ incubator. Quality control strains were incubated for 16–20 hours at 36°C ±1°C in a non-CO_2_ incubator.

### Preparation of agar plates for PAP

Heteroresistance to cefiderocol was tested by the standard PAP method (9). In brief, cefiderocol agar dilution plates with final twofold doubling concentrations of cefiderocol ranging from 0.5 to 64 µg/mL were prepared using MHA following CLSI guidelines (37, 49). Working cefiderocol stock solutions were prepared at 10× the desired stock concentration, then further diluted 1:10 upon addition to the MHA to achieve the desired final concentration. MHA was cooled to 45°C–50°C in a water bath before aseptically adding the cefiderocol solutions. Agar was poured into a Petri dish and allowed to solidify. Antibiotic-free MHA plates were also prepared to serve as growth control plates.

### Determination of heteroresistance by PAP

The presence of heteroresistance was investigated using the PAP methodology described by Sherman et al. for *A. baumannii* (9). PAP susceptibility phenotype was determined once for each CRAB isolate by the central laboratory. All isolates were sub-cultured from freezer stocks on tryptic soy agar plates with 5% sheep blood and incubated at 36°C ± 1°C for 16–20 hours. Inocula were prepared using the broth culture method; a single colony from an overnight plate was used to inoculate a tube containing 1.5 mL of ID-CAMHB. The broth culture was incubated at 36°C ± 1°C until a turbidity equal to 0.5 McFarland (~1.5 × 10^8^ CFU/mL) was reached. Once the desired turbidity was achieved, eight 10-fold serial dilutions in saline were performed, of which seven 10-fold dilutions and undiluted solutions were used.

Each CRAB isolate was serially diluted once and 200 µL of each dilution was added to a 96-well microtiter panel. One set of cefiderocol-containing agar plates and a growth plate were inoculated in triplicate with 10 µL per spot (containing approximately 1.5 × 10^6^ CFU) taken from each dilution well and undiluted well using the Integra Viaflo liquid handler (Integra Biosciences Corp., Hudson, NH, USA). Inocula were allowed to absorb into the agar plates for 10 minutes. Plates were covered, inverted, and placed in a non-CO_2_ incubator at 36°C ± 1°C for 72 hours. Each plate was removed from the incubator to be read at 24, 48, and 72 hours, and the number of colonies per spot was counted as CFU.

### Determination of colony counts

For all replicates of each CRAB isolate at each time point, the CFU/mL of the plate containing no cefiderocol was recorded as the viable count of the original inoculum. CFUs from cefiderocol-containing agar plates were determined for cefiderocol concentrations ranging from 0.5 to 64 µg/mL. The number of colonies per spot was recorded for the highest dilution factor at which there was growth. If growth was detected, spots with between 3 and 30 discrete colonies were enumerated and recorded for each plate. Confluent growth or growth with greater than 30 colonies was recorded as uncountable and recorded as 5 × 10^10^ CFU/mL; spots with no growth or fewer than three colonies per spot were recorded as 1 × 10^0^ CFU/mL to allow for the plotting of growth charts.

### Categorization of PAP

Categorization of isolates as susceptible, resistant, or heteroresistant by the PAP method was carried out according to the definitions recommended by Sherman et al. (9) and were consistent with those used in the paper by Choby et al. (18). The colony counts at each cefiderocol concentration and time point were analyzed, and the proportion of surviving cells was determined as the ratio of “number of colonies per spot on cefiderocol plate × dilution factor” /“number of colonies per spot on antibiotic-free plate × dilution factor”.

The interpretation of the ratios for surviving cells was used to categorize the isolates as susceptible, heteroresistant, or resistant in the PAP test. The cefiderocol CLSI resistance breakpoint based on the BMD method using ID-CAMHB is 16 µg/mL; thus, 4× the resistance breakpoint (i.e., 64 µg/mL) was selected as the highest cefiderocol concentration in the agar plates that could be used for the determination of the isolates as susceptible, heteroresistant, or resistant by PAP methodology, as defined previously (9, 23). Susceptible isolates were defined as those in which the proportion of surviving bacteria at 16–64 µg/mL (equal to or greater than the CLSI breakpoint of 16 µg/mL) was <10^–7^ (23). Resistance was defined as isolates in which the proportion of survivors at concentrations of either 16, 32, or 64 µg/mL was >0.5 (>5 × 10^–1^) (23). Heteroresistant isolates were defined as those where the proportion of surviving bacteria at 64 µg/mL (i.e., 4× the CLSI resistance breakpoint) was ≥10^–7^ but ≤0.5 (9, 23). The final phenotypic category was considered as the phenotype defined at the 72-hour time point.

### Outcomes

Heteroresistance to cefiderocol by PAP, clinical and microbiological outcomes at EOT (last day of treatment), TOC [end of treatment + 7 days (±2 days)], and follow-up, and ACM at Day 14, Day 28, and EOS [end of treatment + 28 (±3) days], and association of PAP-phenotypes to these study outcomes were analyzed for patients with CRAB. Definitions of clinical and microbiological outcomes are described elsewhere (7). Molecular characterization of the isolates (7) and at least fourfold increases in cefiderocol MIC were determined previously (7, 48).

### Statistical analysis

Descriptive statistics by PAP phenotype were used for the analysis of baseline characteristics, clinical and microbiological outcomes, and ACM.

## Data Availability

All analyzed data are included in the manuscript. Upon reasonable request, further data may be available from the corresponding author.

## SUPPLEMENTAL MATERIAL

Figures S1.1 to S1.38 spectrum.02371-23-s0001.pdf Growth profiles of 38 carbapenem-resistant *Acinetobacter calcoaceticus-baumannii* complex isolates from the CREDIBLE-CR study by PAP method.

Table S1 spectrum.02371-23-s0002.pdf Clinical characteristics of patients with carbapenem-resistant *Acinetobacter calcoaceticus-baumannii* complex who died or survived by end of study in the cefiderocol arm in the CREDIBLE-CR study.

ASM does not own the copyrights to Supplemental Material that may be linked to, or accessed through, an article. The authors have granted ASM a non-exclusive, world-wide license to publish the Supplemental Material files. Please contact the corresponding author directly for reuse.
