# Supplementary material for: Heteroresistance to cefiderocol in carbapenem-resistant Acinetobacter baumannii in the CREDIBLE-CR study was not linked to clinical outcomes: a post hoc analysis
Source: Microbiol Spectr. 2023 Nov 15;11(6):e02371-23. doi: 10.1128/spectrum.02371-23 (PMC10714777; doi:10.1128/spectrum.02371-23)
Supplement: Figures S1.1 to S1.38 — Growth profiles of 38 carbapenem-resistant Acinetobacter calcoaceticus-baumannii complex isolates from the CREDIBLE-CR study by PAP method. [file spectrum.02371-23-s0001.pdf]

Heteroresistance to cefiderocol in carbapenem-resistant *Acinetobacter baumannii* in the CREDIBLE-CR study was not linked to clinical outcomes: a post-hoc analysis

Christopher Longshaw, Anne Santerre Henriksen, Dana Dressel, Michelle Malysa, Christian Silvestri, Miki Takemura, Yoshinori Yamano, Takamichi Baba, Christine M Slover

Supplementary Figures

Supplementary Figures S1.1–S1.38. Growth profiles of 38 carbapenem-resistant *Acinetobacter calcoaceticus-baumannii* complex isolates from the CREDIBLE-CR study by PAP method

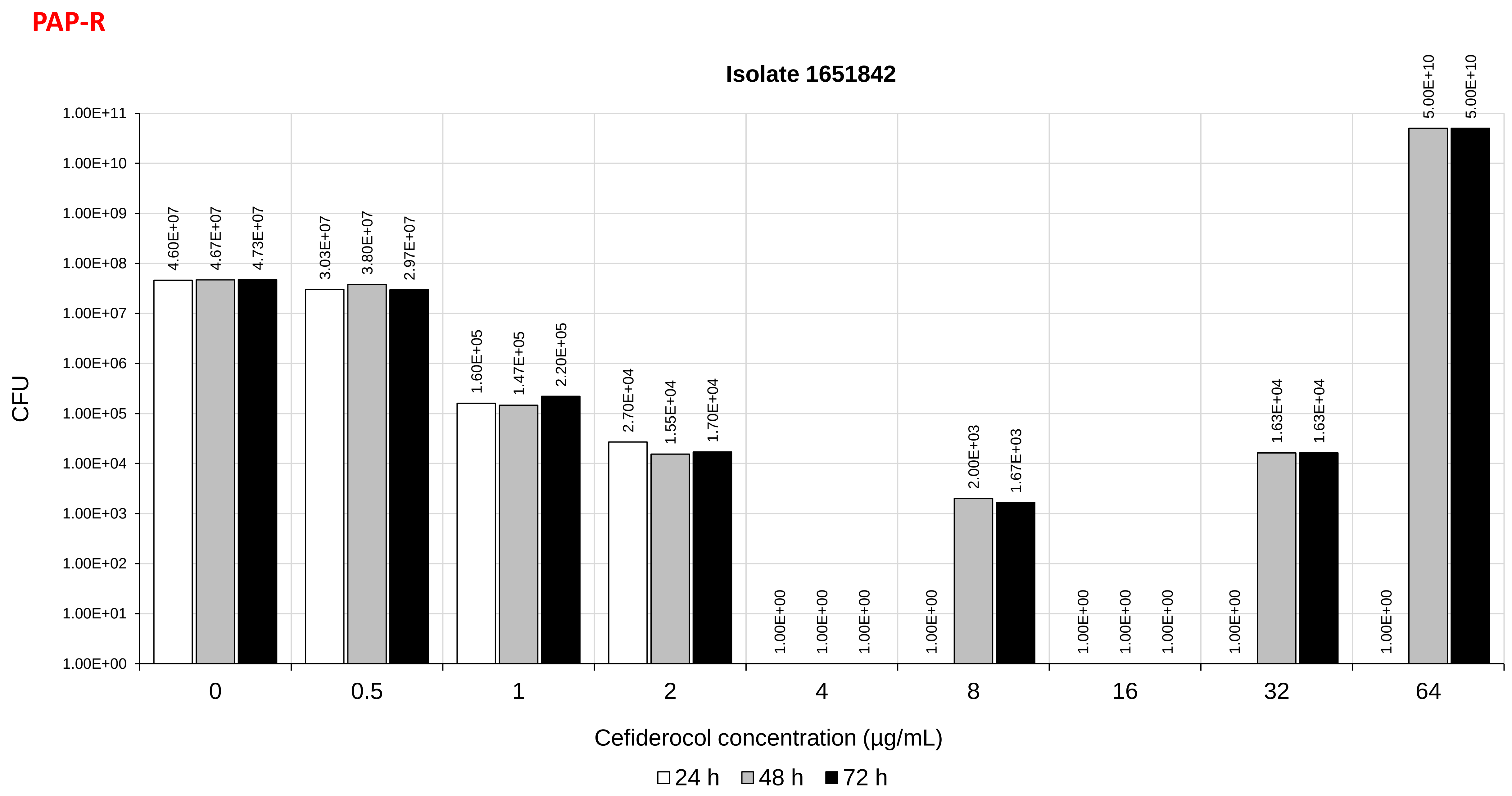

CFU, colony-forming unit; PAP, population analysis profiling; R, resistant.

Supplementary Fig S1.1.
Growth profile of carbapenem-resistant *Acinetobacter calcoaceticus-baumannii* complex by PAP method

PAP-HR

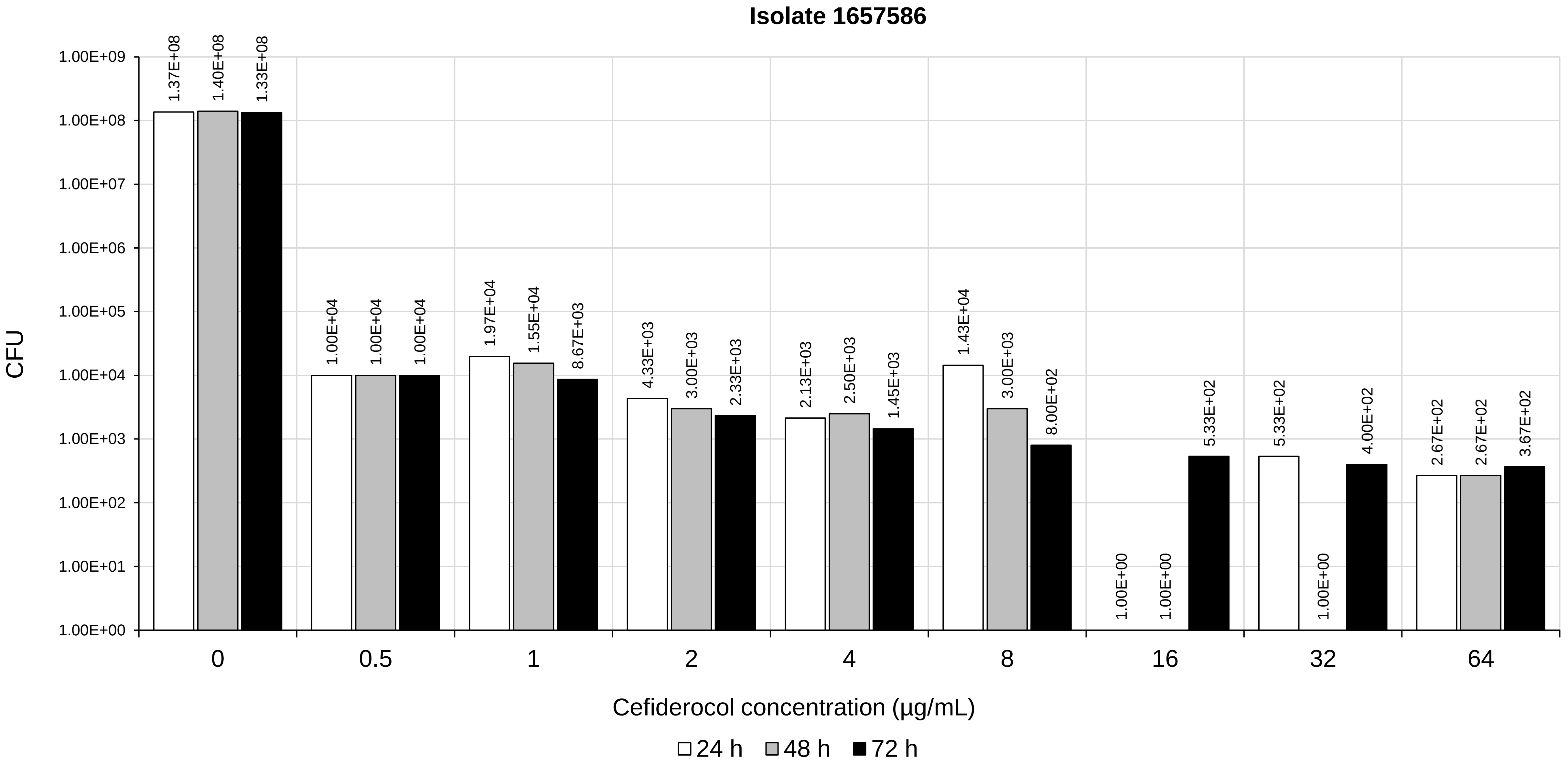

CFU, colony-forming unit; HR, heteroresistant; PAP, population analysis profiling.

Supplementary Fig S1.2.
Growth profile of carbapenem-resistant *Acinetobacter calcoaceticus-baumannii* complex by PAP method

PAP-HR

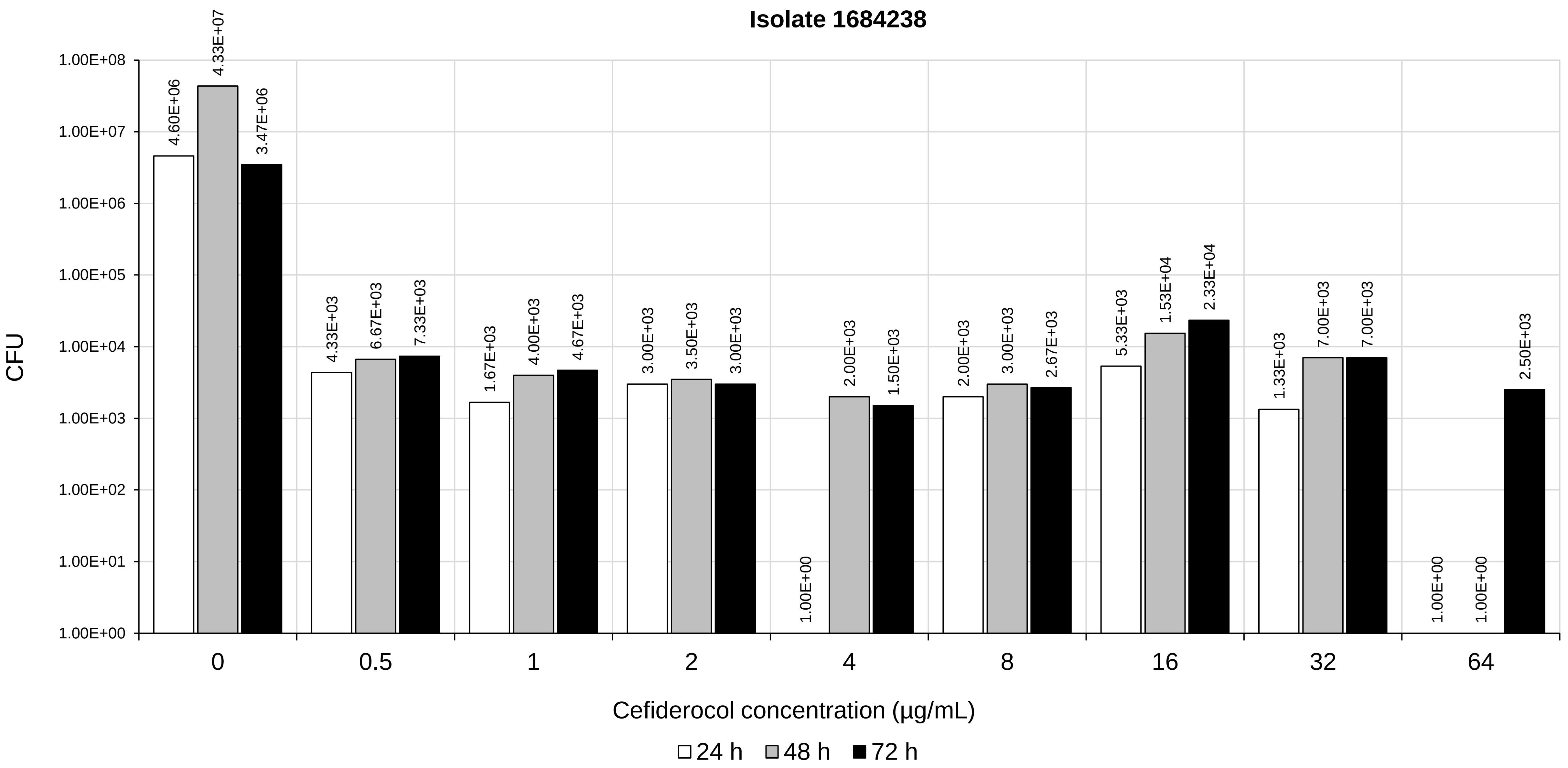

CFU, colony-forming unit; HR, heteroresistant; PAP, population analysis profiling.

Supplementary Fig S1.3.
Growth profile of carbapenem-resistant *Acinetobacter calcoaceticus-baumannii* complex by PAP method

PAP-HR

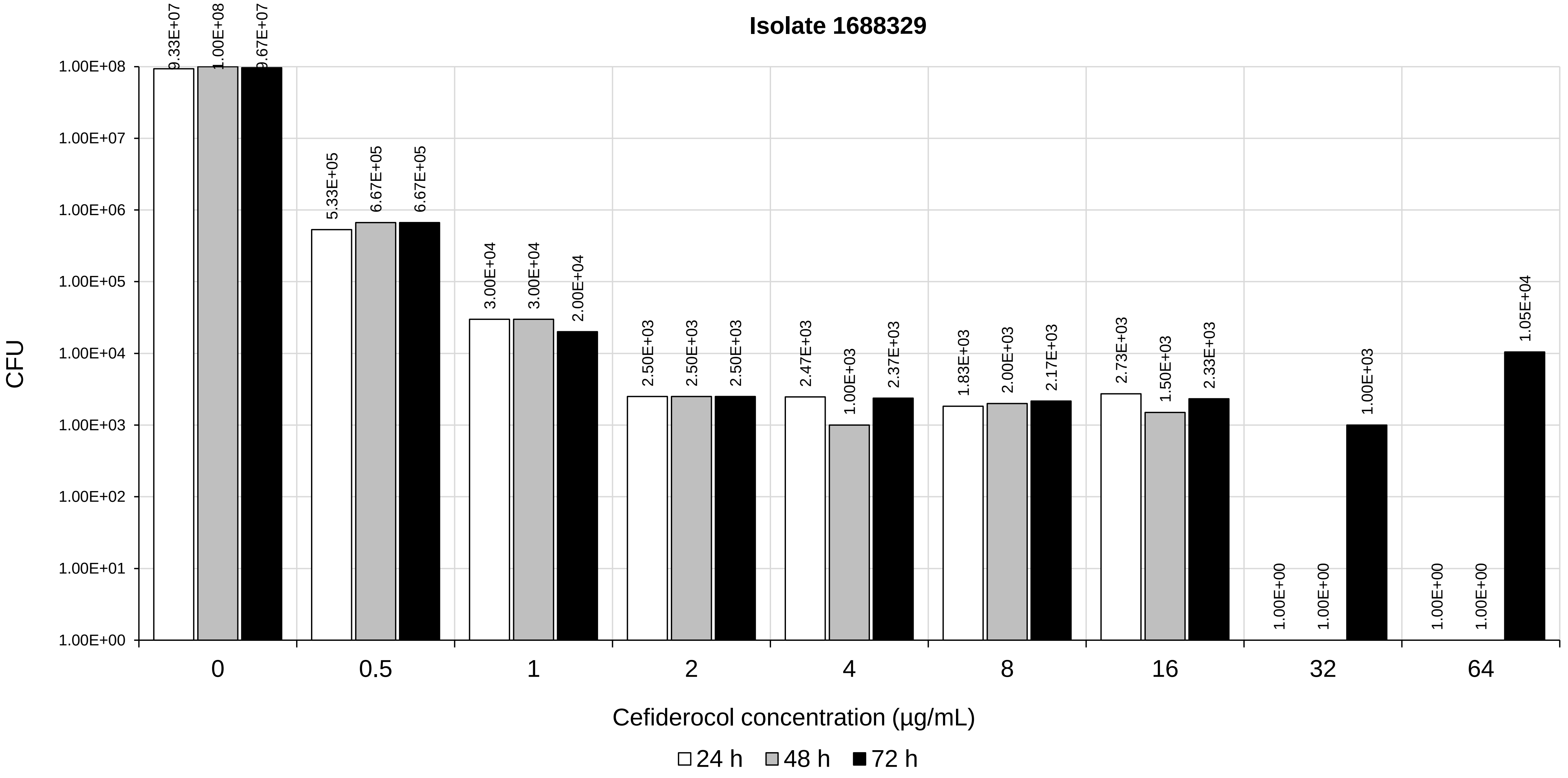

CFU, colony-forming unit; HR, heteroresistant; PAP, population analysis profiling.

Supplementary Fig S1.4.
Growth profile of carbapenem-resistant *Acinetobacter calcoaceticus-baumannii* complex by PAP method

PAP-R

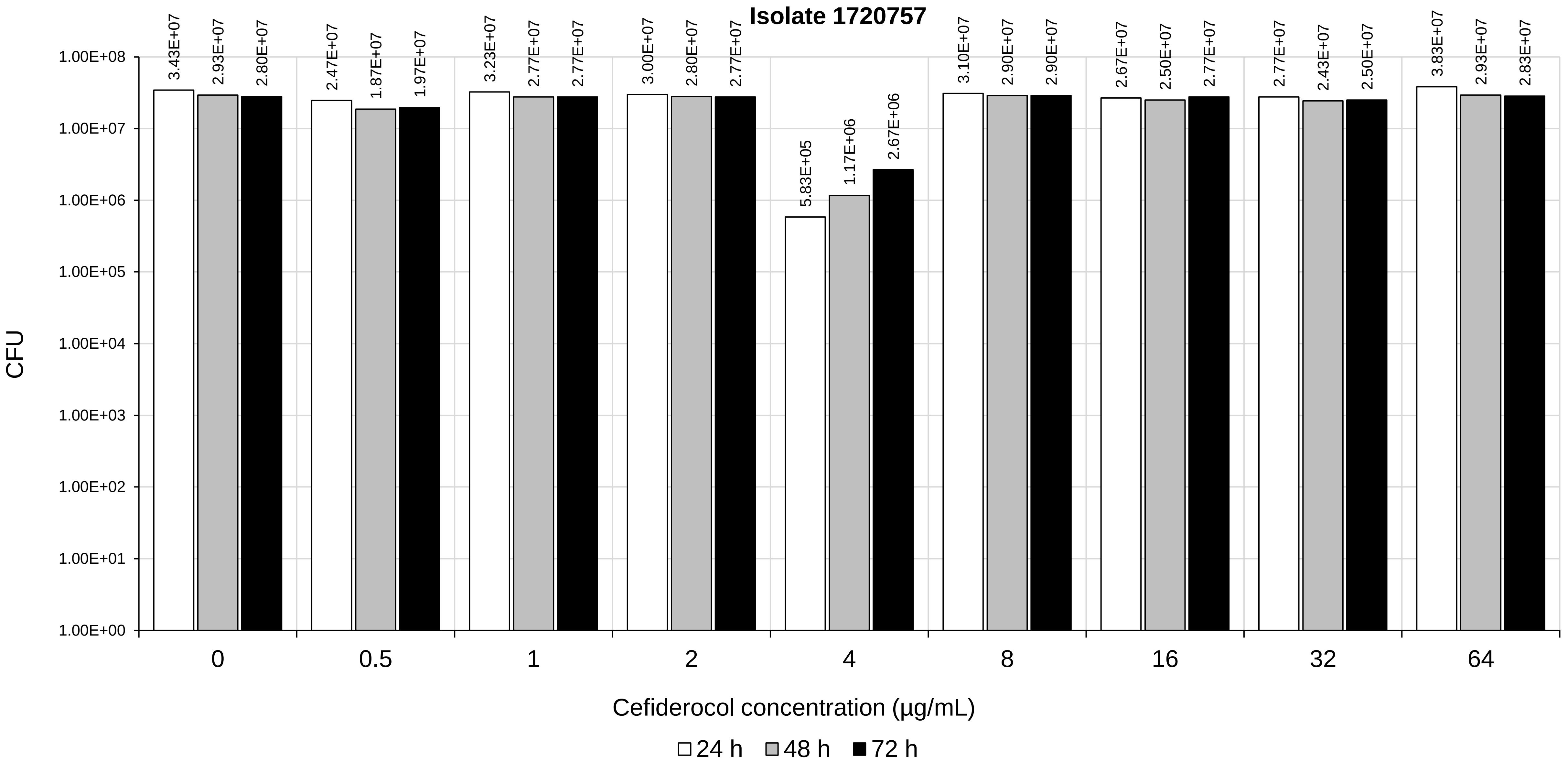

CFU, colony-forming unit; PAP, population analysis profiling; R, resistant.

Supplementary Fig S1.5.
Growth profile of carbapenem-resistant *Acinetobacter calcoaceticus-baumannii* complex by PAP method

PAP-HR

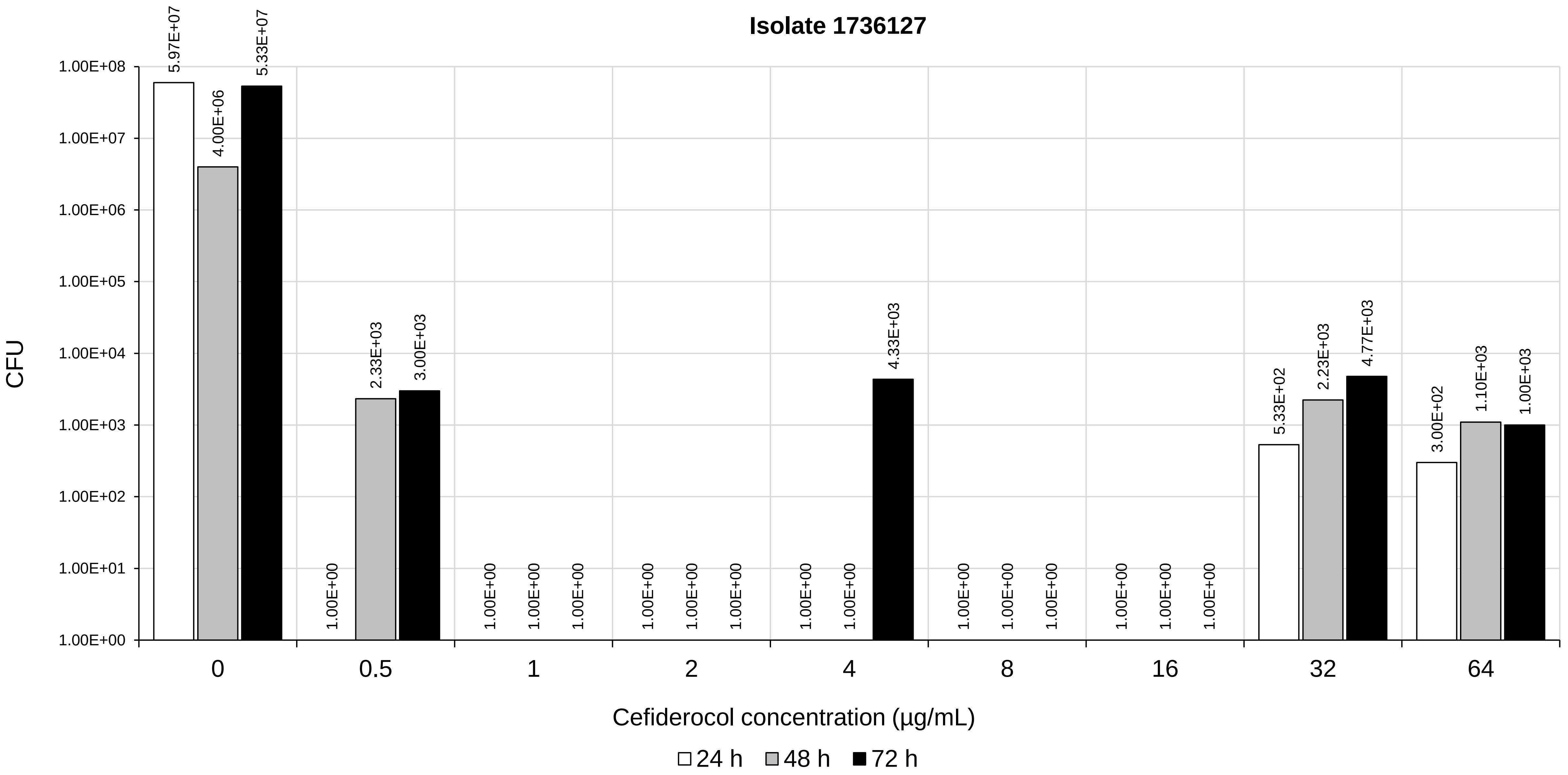

CFU, colony-forming unit; HR, heteroresistant; PAP, population analysis profiling.

Supplementary Fig S1.6.
Growth profile of carbapenem-resistant *Acinetobacter calcoaceticus-baumannii* complex by PAP method

PAP-HR

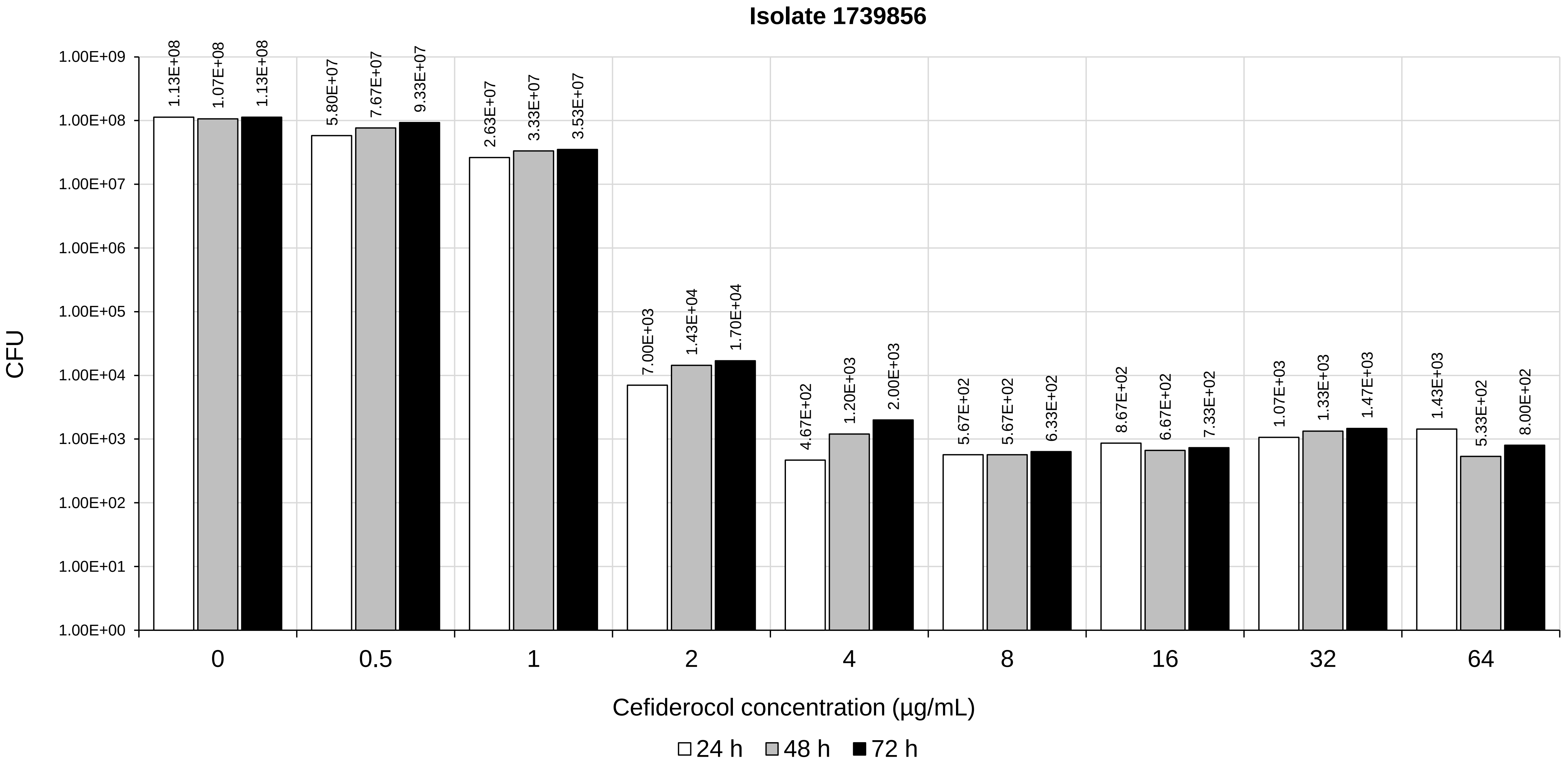

CFU, colony-forming unit; HR, heteroresistant; PAP, population analysis profiling.

Supplementary Fig S1.7.
Growth profile of carbapenem-resistant *Acinetobacter calcoaceticus-baumannii* complex by PAP method

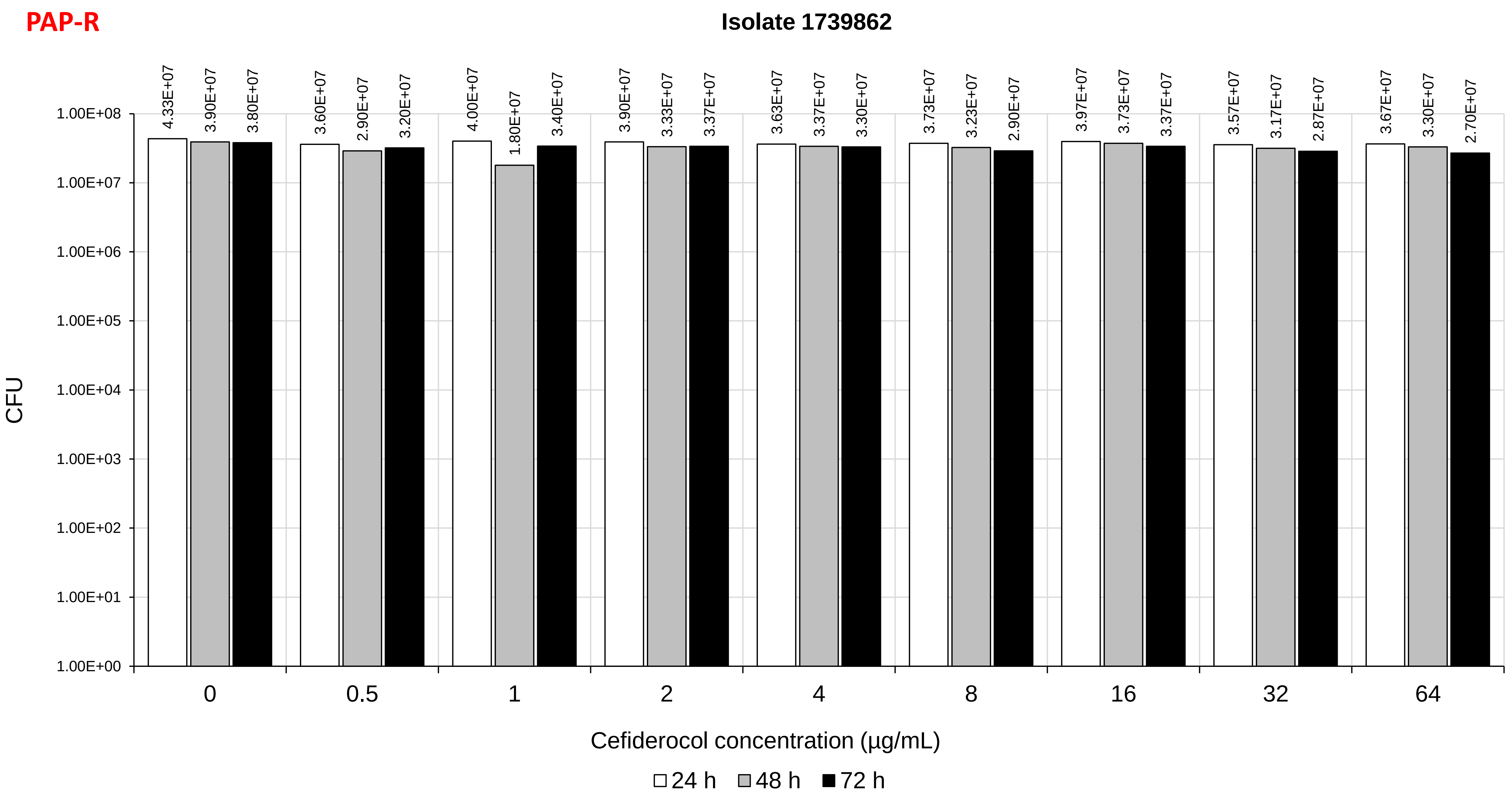

CFU, colony-forming unit; PAP, population analysis profiling; R, resistant.

Supplementary Fig S1.8.
Growth profile of carbapenem-resistant *Acinetobacter calcoaceticus-baumannii* complex by PAP method

PAP-HR

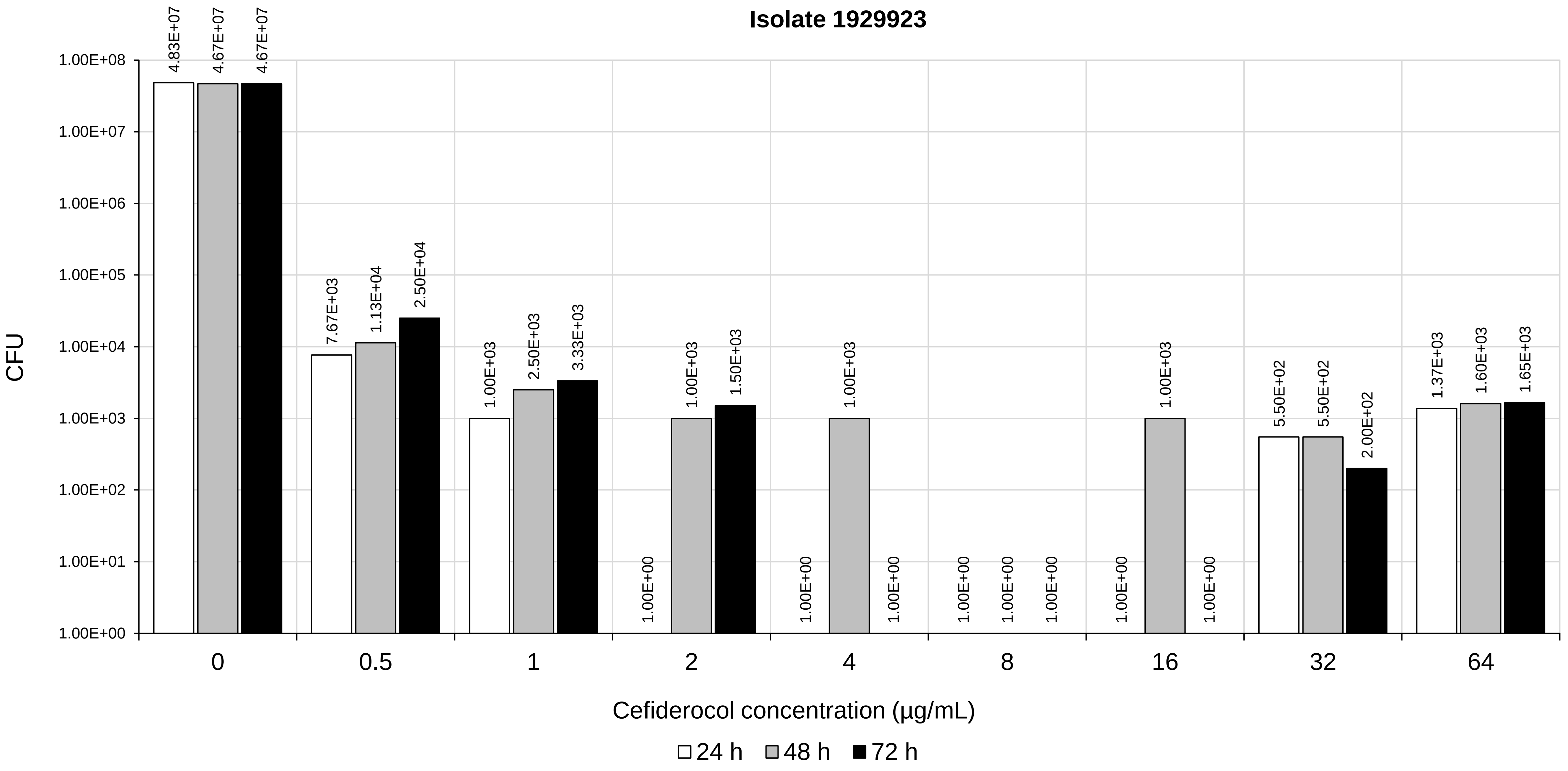

CFU, colony-forming unit; HR, heteroresistant; PAP, population analysis profiling.

Supplementary Fig S1.9.
Growth profile of carbapenem-resistant *Acinetobacter calcoaceticus-baumannii* complex by PAP method

PAP-S

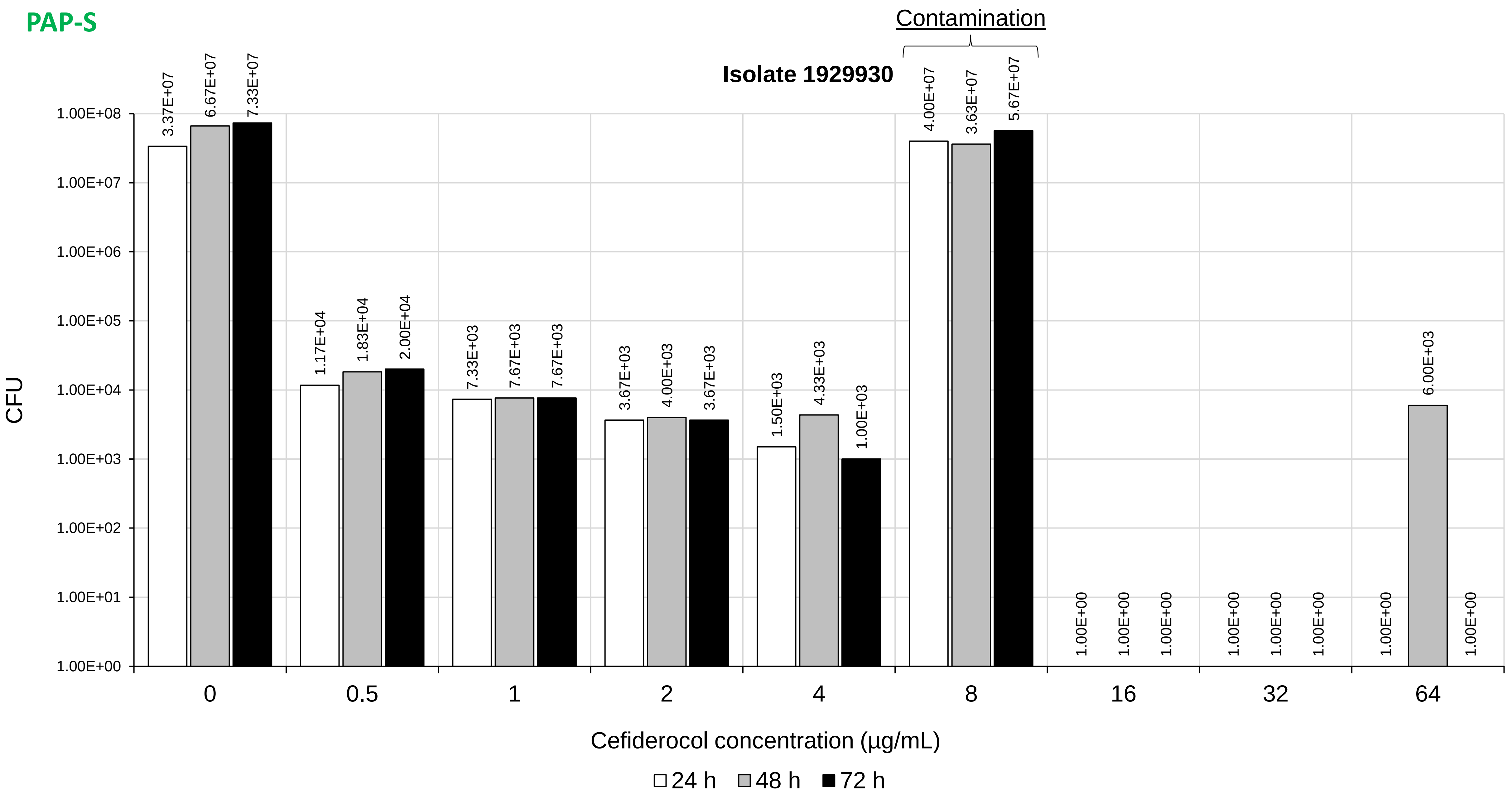

CFU, colony-forming unit; PAP, population analysis profiling; S, susceptible.

Supplementary Fig S1.10.
Growth profile of carbapenem-resistant *Acinetobacter calcoaceticus-baumannii* complex by PAP method

PAP-S

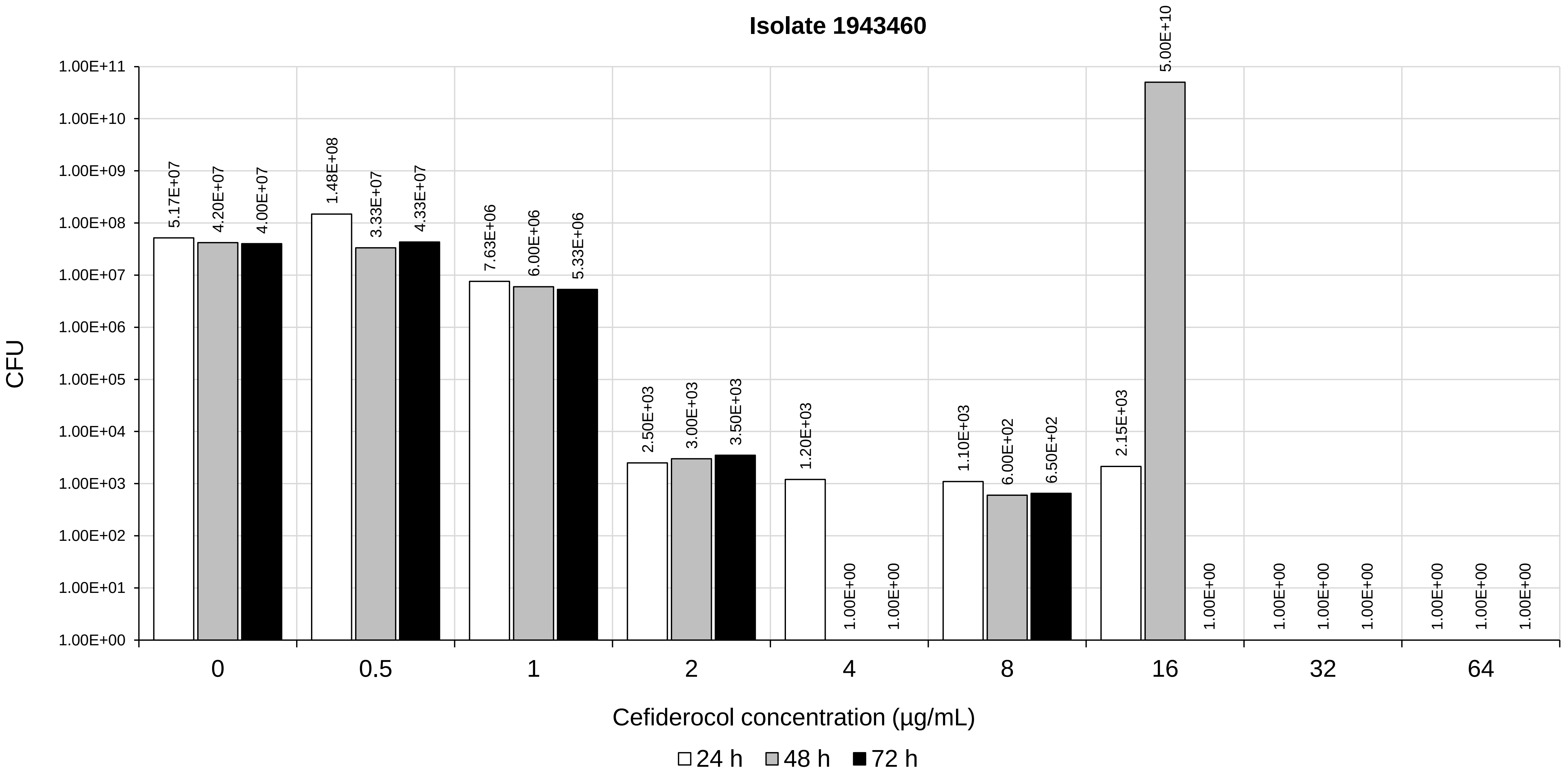

CFU, colony-forming unit; PAP, population analysis profiling; S, susceptible.

Supplementary Fig S1.11.
Growth profile of carbapenem-resistant *Acinetobacter calcoaceticus-baumannii* complex by PAP method

PAP-S

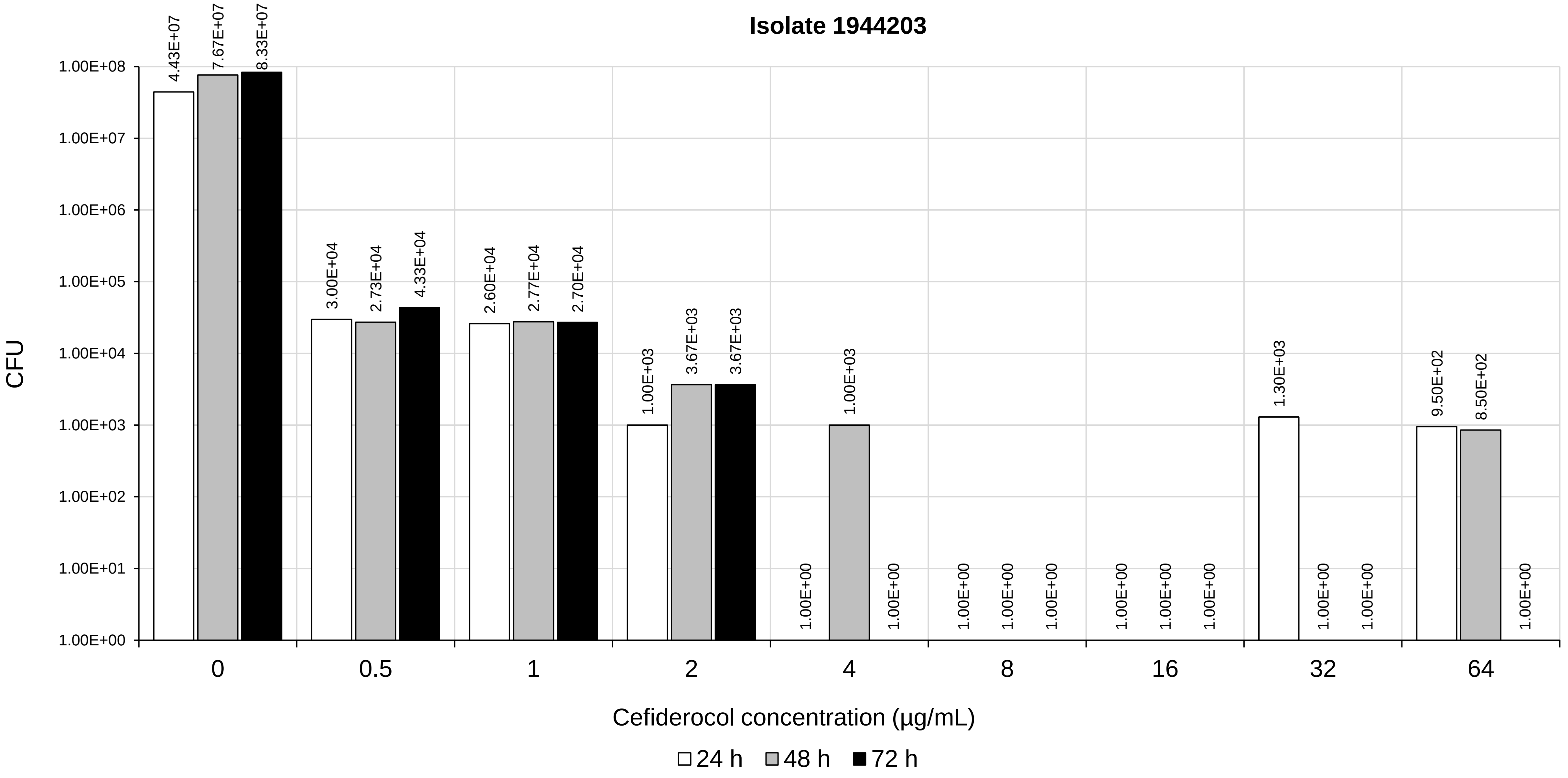

CFU, colony-forming unit; PAP, population analysis profiling; S, susceptible.

Supplementary Fig S1.12.
Growth profile of carbapenem-resistant *Acinetobacter calcoaceticus-baumannii* complex by PAP method

PAP-HR

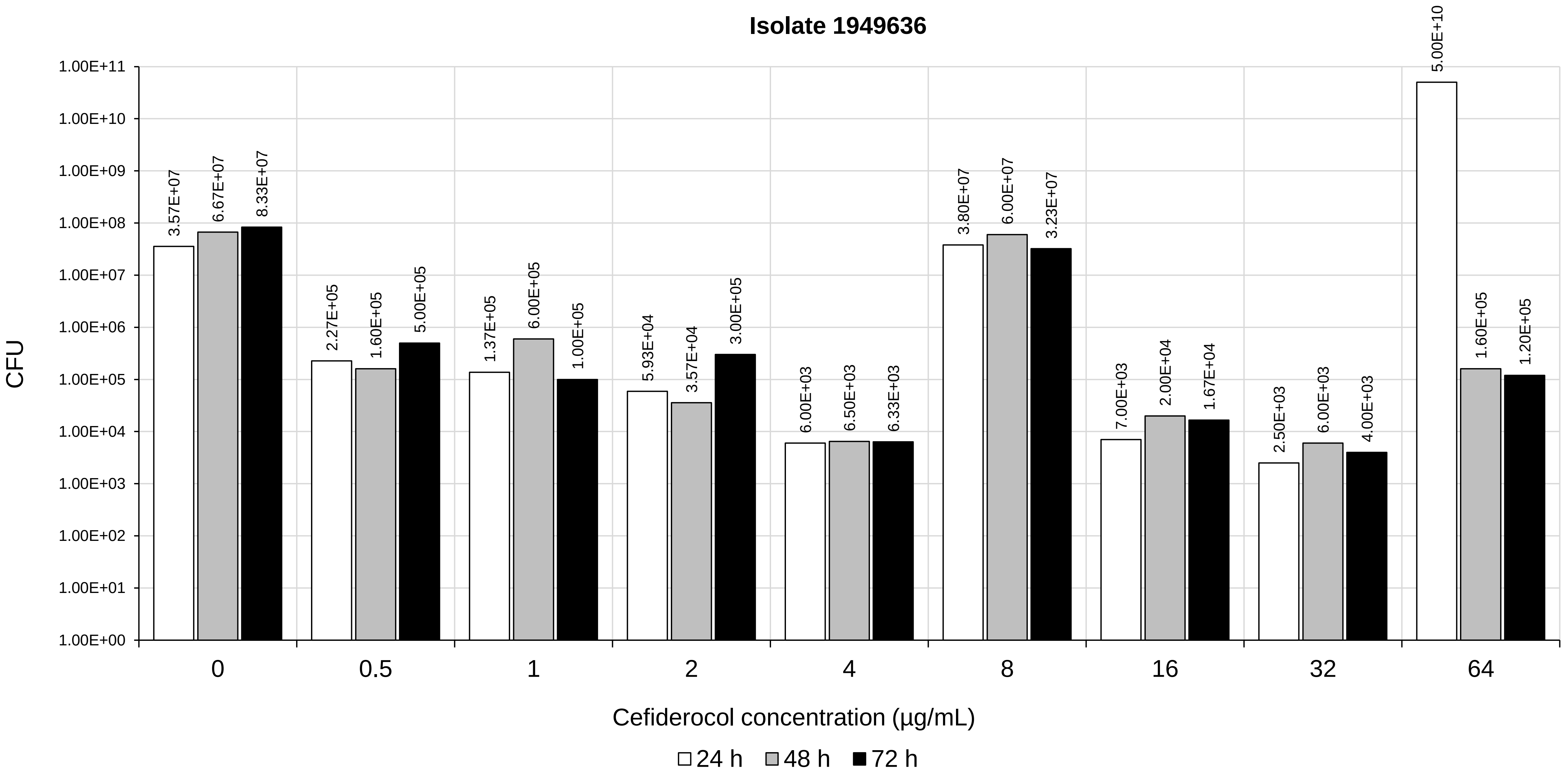

CFU, colony-forming unit; HR, heteroresistant; PAP, population analysis profiling.

Supplementary Fig S1.13.
Growth profile of carbapenem-resistant *Acinetobacter calcoaceticus-baumannii* complex by PAP method

PAP-HR

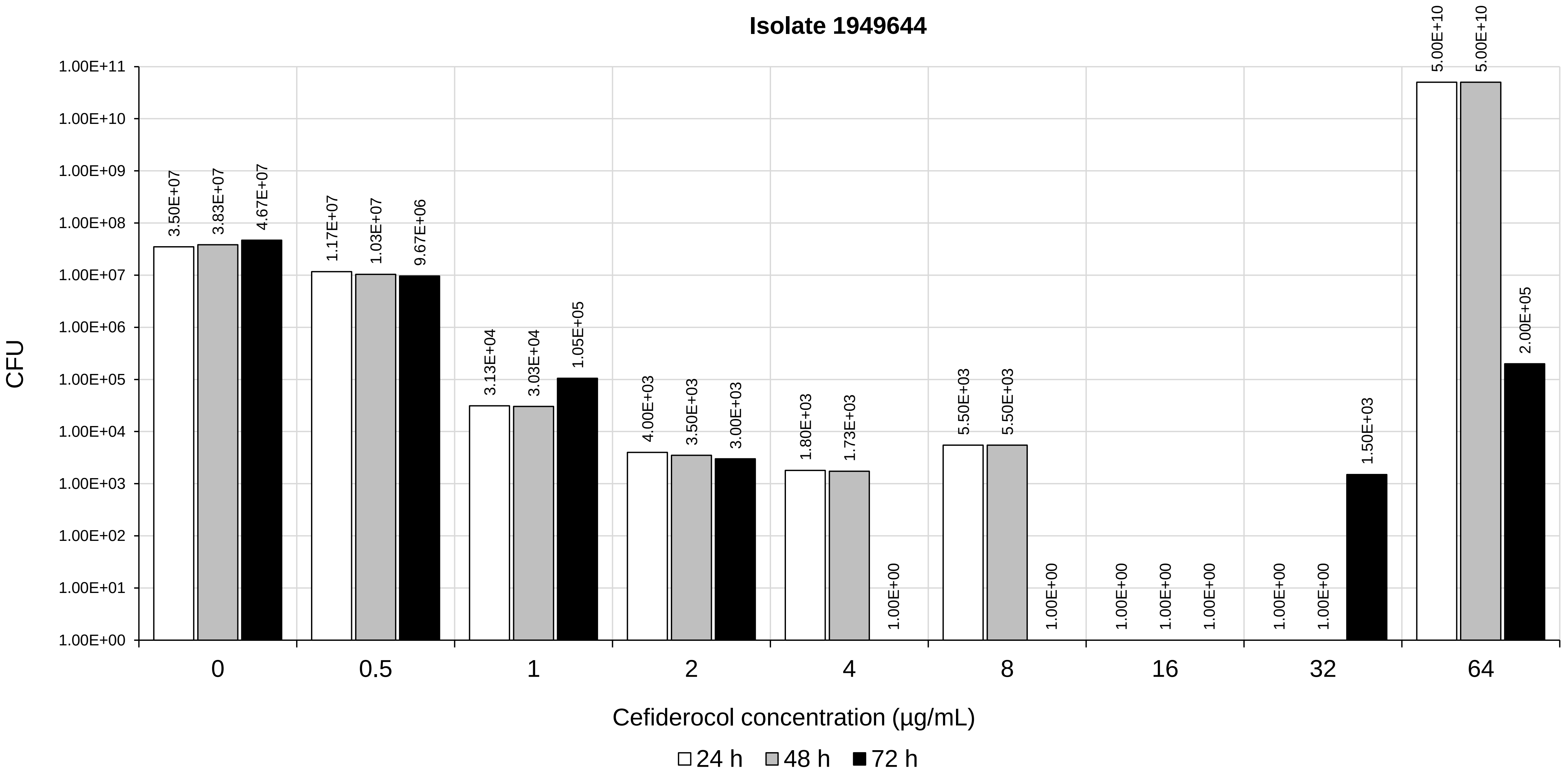

CFU, colony-forming unit; HR, heteroresistant; PAP, population analysis profiling.

Supplementary Fig S1.14.
Growth profile of carbapenem-resistant *Acinetobacter calcoaceticus-baumannii* complex by PAP method

PAP-R

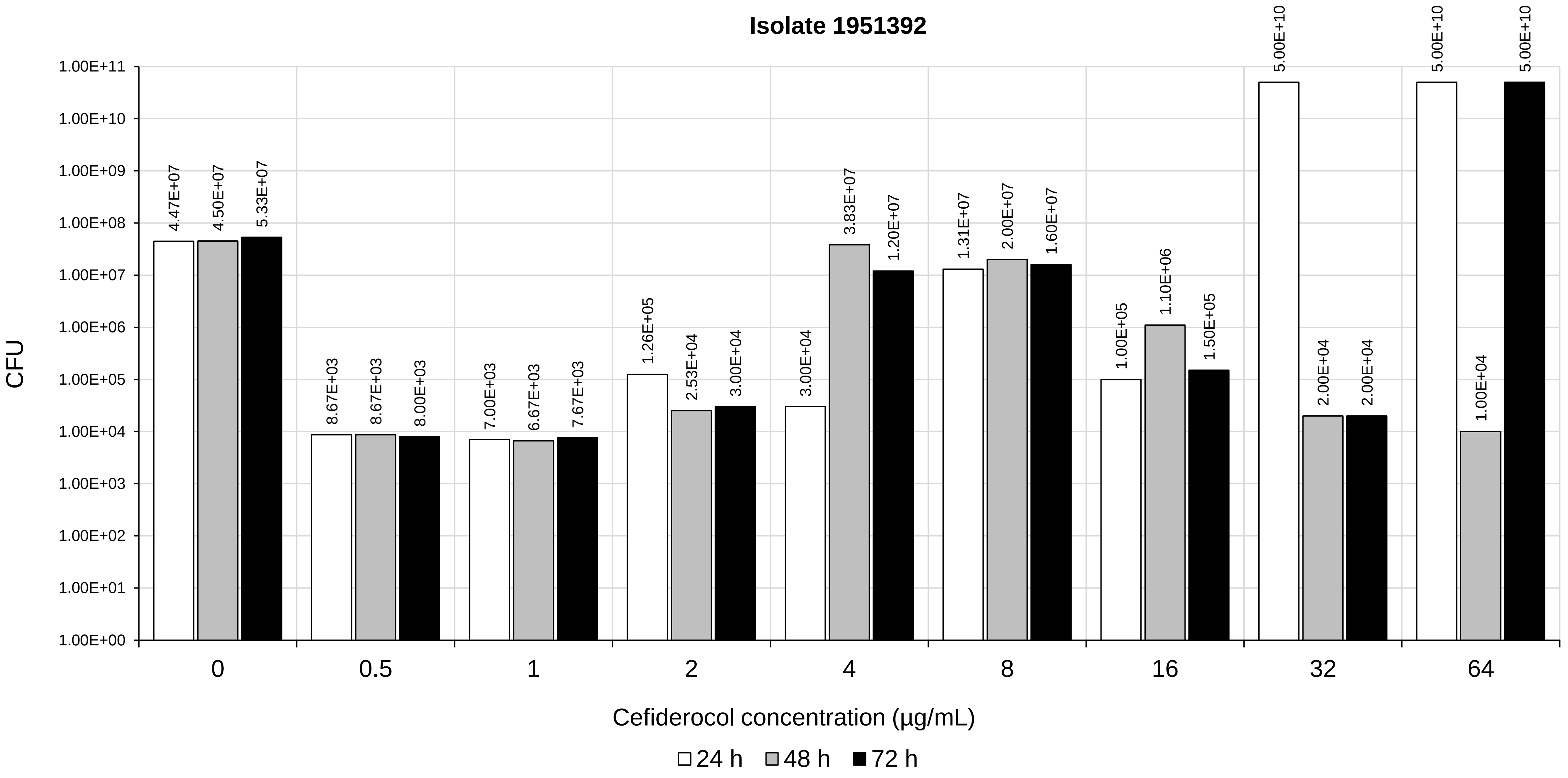

CFU, colony-forming unit; PAP, population analysis profiling; R, resistant.

Supplementary Fig S1.15.
Growth profile of carbapenem-resistant *Acinetobacter calcoaceticus-baumannii* complex by PAP method

PAP-HR

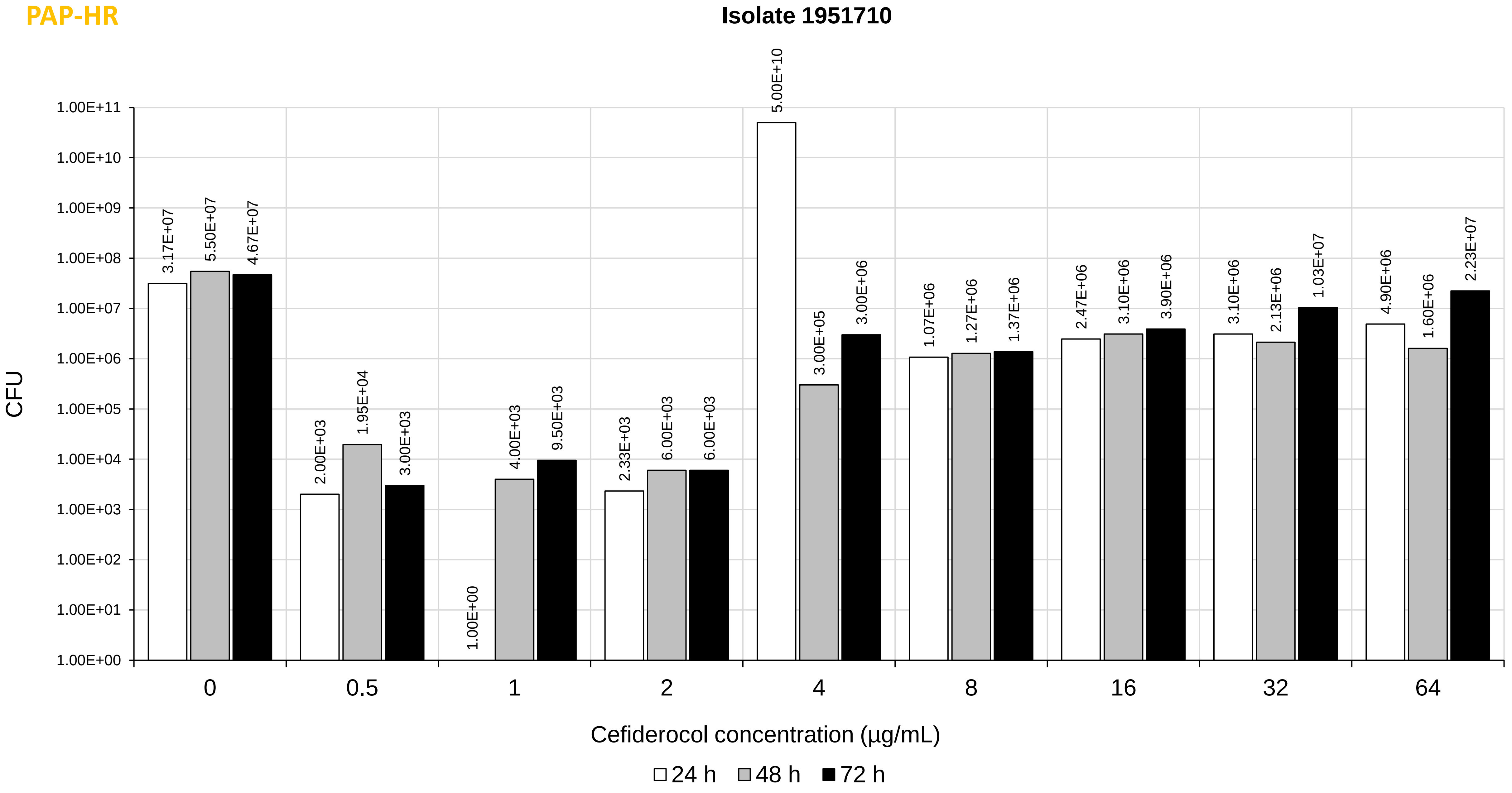

CFU, colony-forming unit; HR, heteroresistant; PAP, population analysis profiling.

Supplementary Fig S1.16.
Growth profile of carbapenem-resistant *Acinetobacter calcoaceticus-baumannii* complex by PAP method

PAP-R

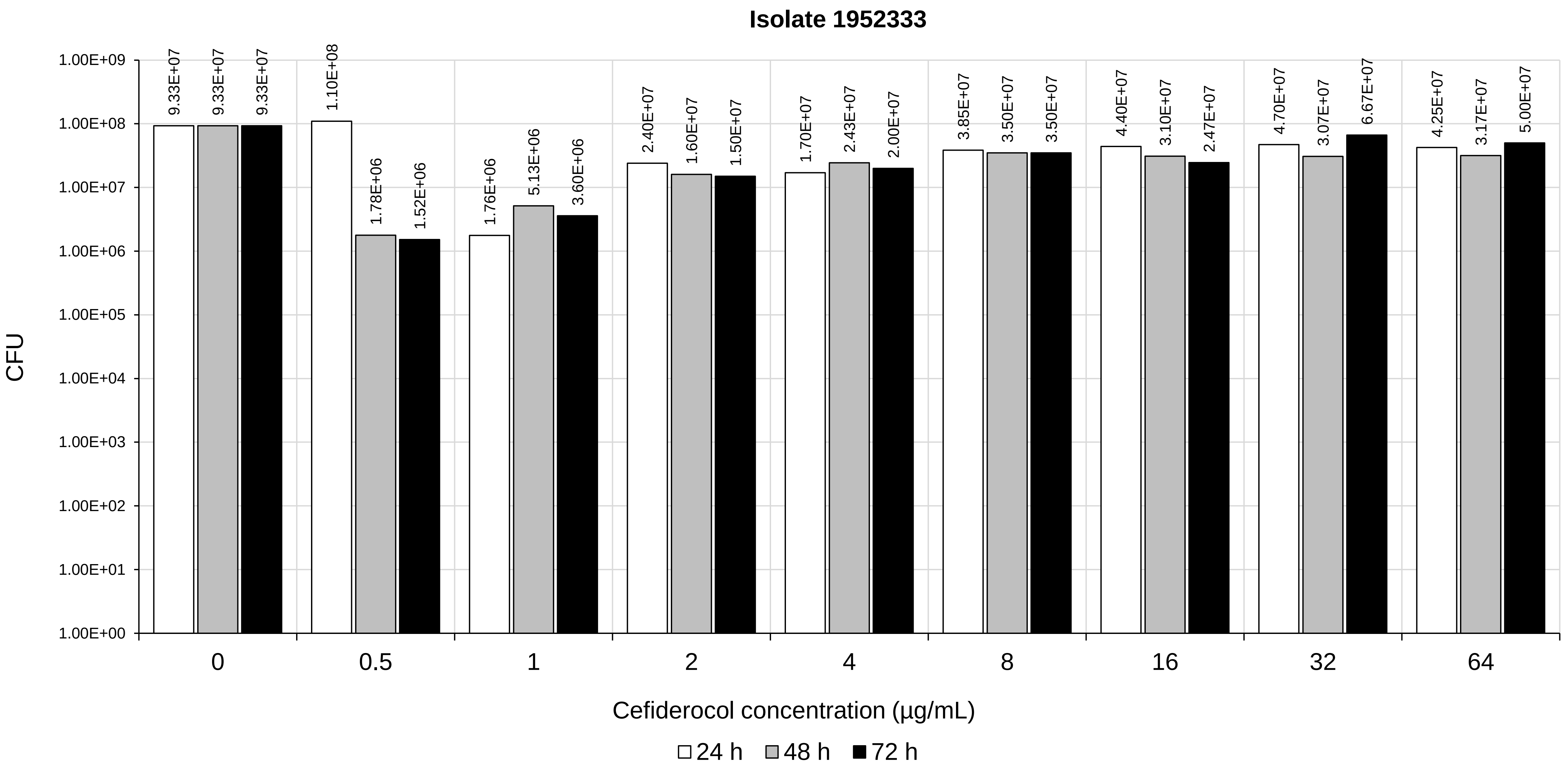

CFU, colony-forming unit; PAP, population analysis profiling; R, resistant.

Supplementary Fig S1.17.
Growth profile of carbapenem-resistant *Acinetobacter calcoaceticus-baumannii* complex by PAP method

PAP-HR

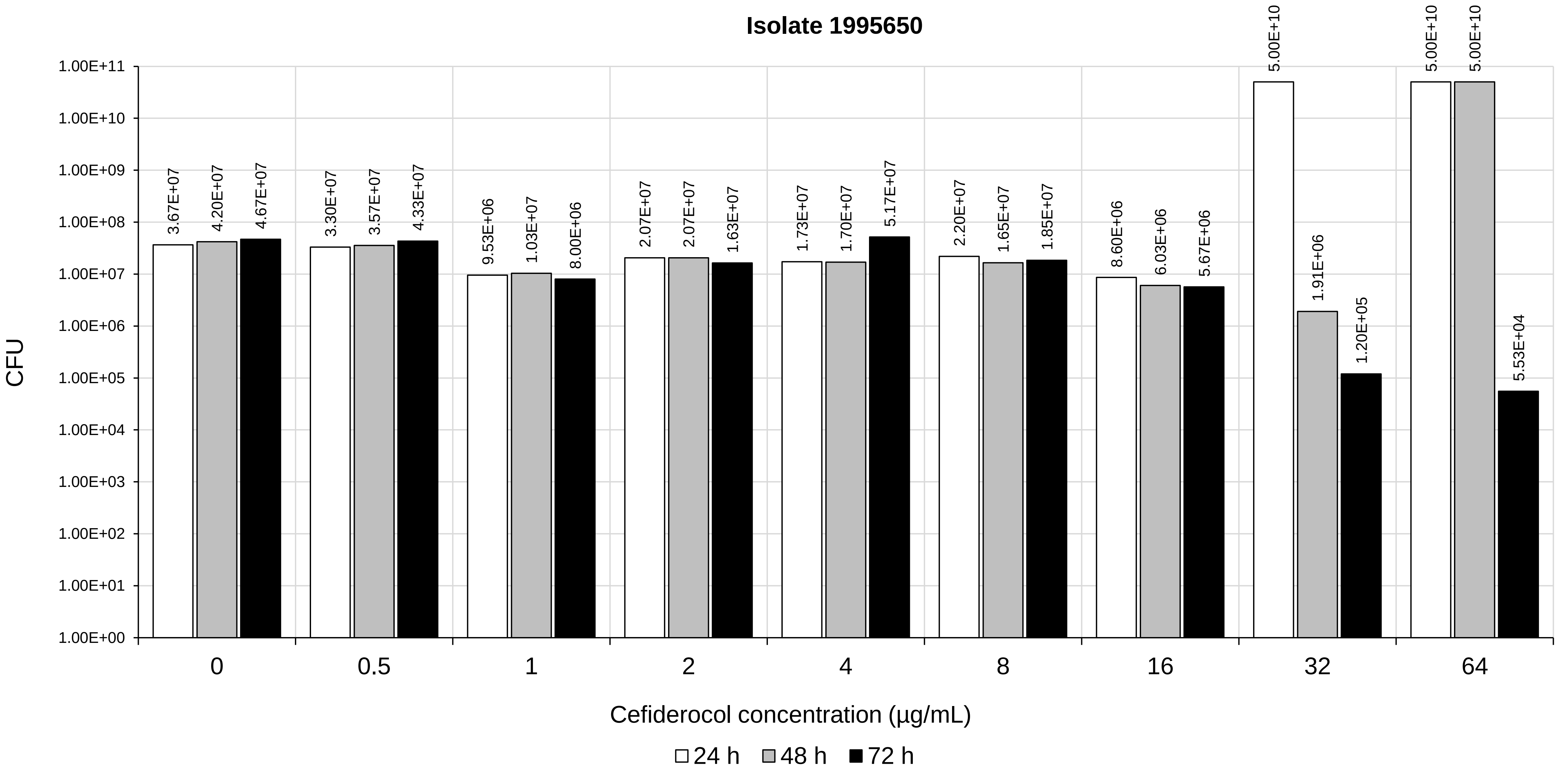

CFU, colony-forming unit; HR, heteroresistant; PAP, population analysis profiling.

Supplementary Fig S1.18.
Growth profile of carbapenem-resistant *Acinetobacter calcoaceticus-baumannii* complex by PAP method

PAP-S

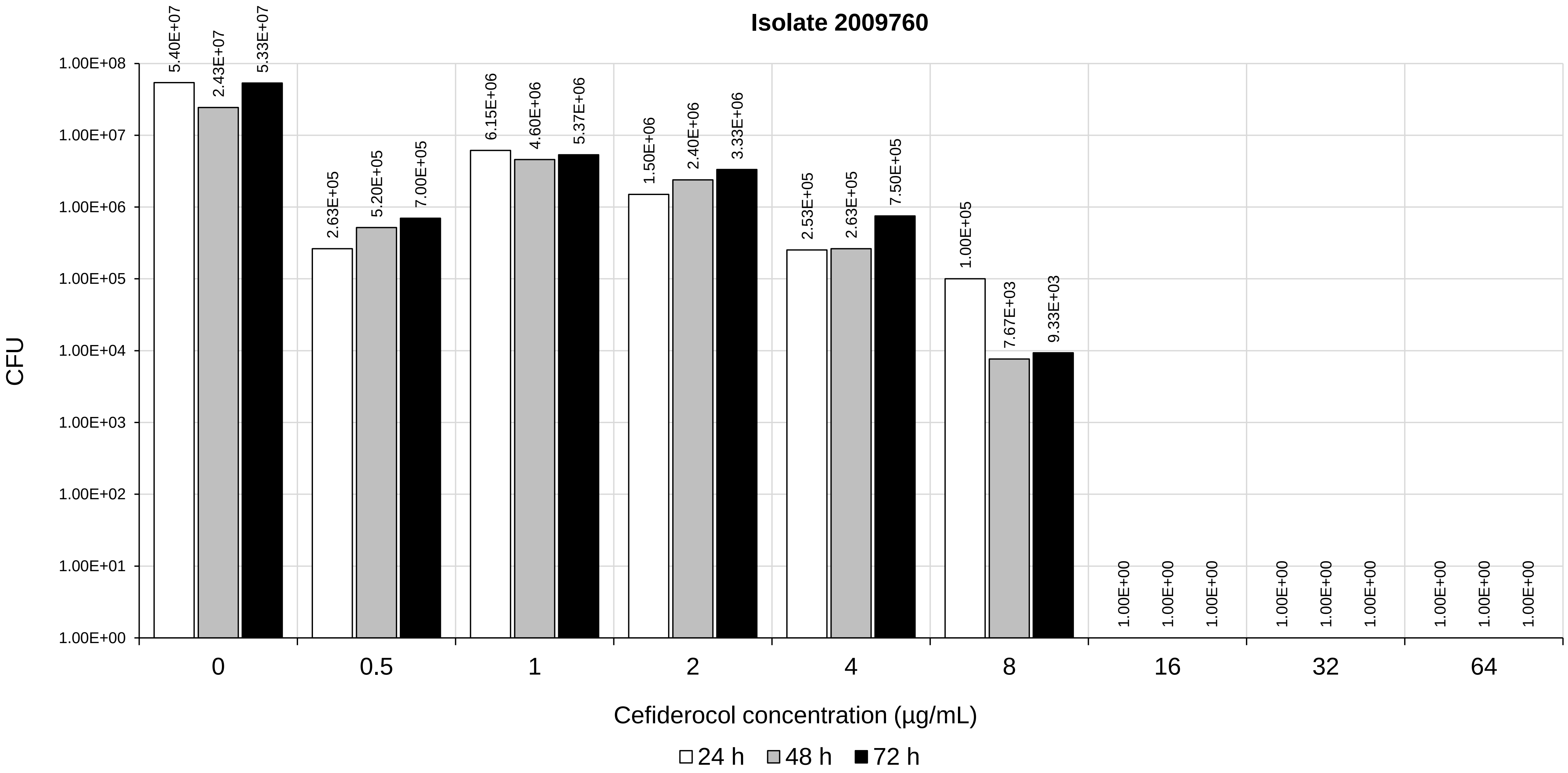

CFU, colony-forming unit; PAP, population analysis profiling; S, susceptible.

Supplementary Fig S1.19.
Growth profile of carbapenem-resistant *Acinetobacter calcoaceticus-baumannii* complex by PAP method

PAP-HR

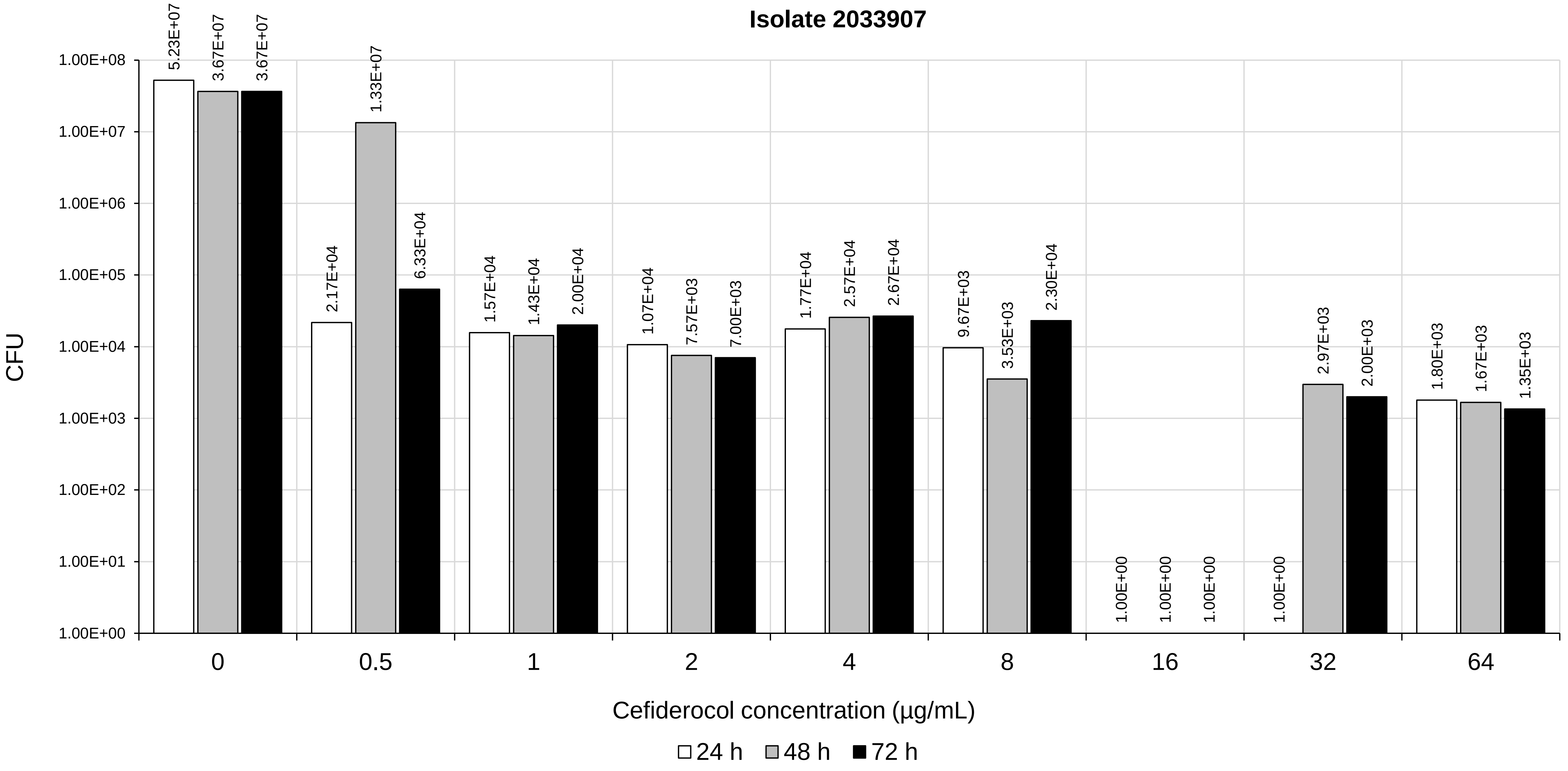

CFU, colony-forming unit; HR, heteroresistant; PAP, population analysis profiling.

Supplementary Fig S1.20.
Growth profile of carbapenem-resistant *Acinetobacter calcoaceticus-baumannii* complex by PAP method

PAP-R

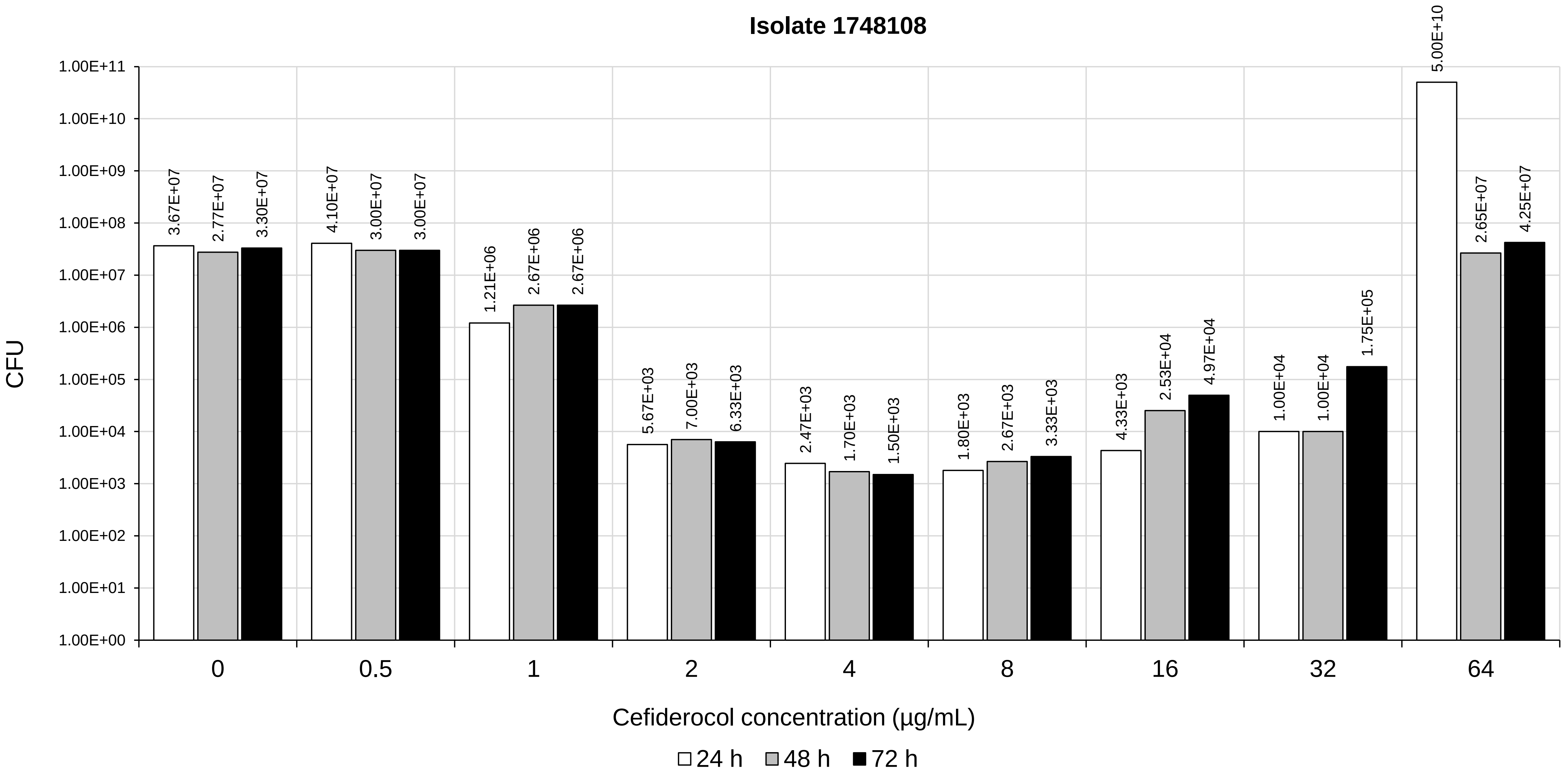

CFU, colony-forming unit; PAP, population analysis profiling; R, resistant.

Supplementary Fig S1.21.
Growth profile of carbapenem-resistant *Acinetobacter calcoaceticus-baumannii* complex by PAP method

PAP-R

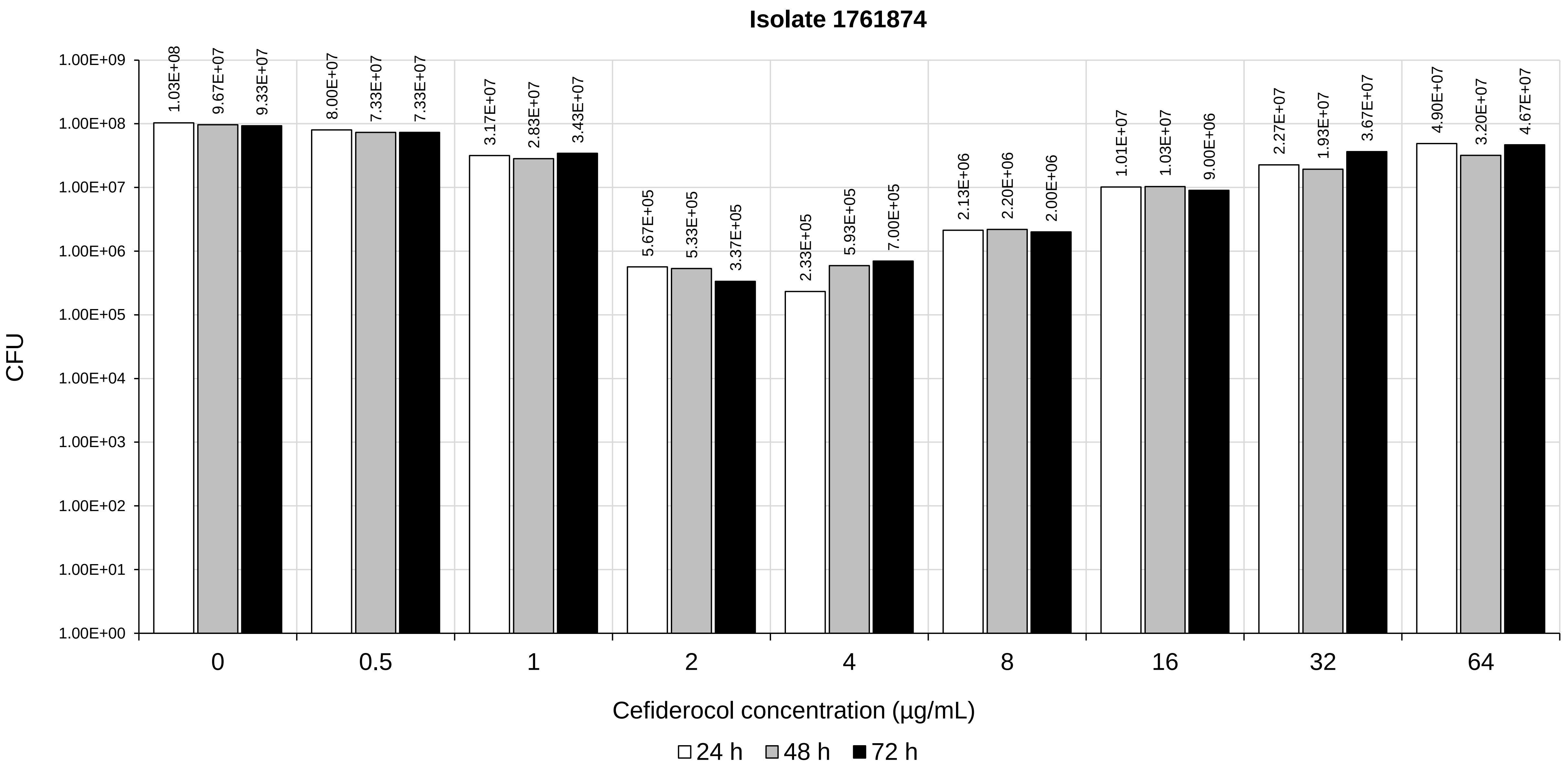

CFU, colony-forming unit; PAP, population analysis profiling; R, resistant.

Supplementary Fig S1.22.
Growth profile of carbapenem-resistant *Acinetobacter calcoaceticus-baumannii* complex by PAP method

PAP-HR

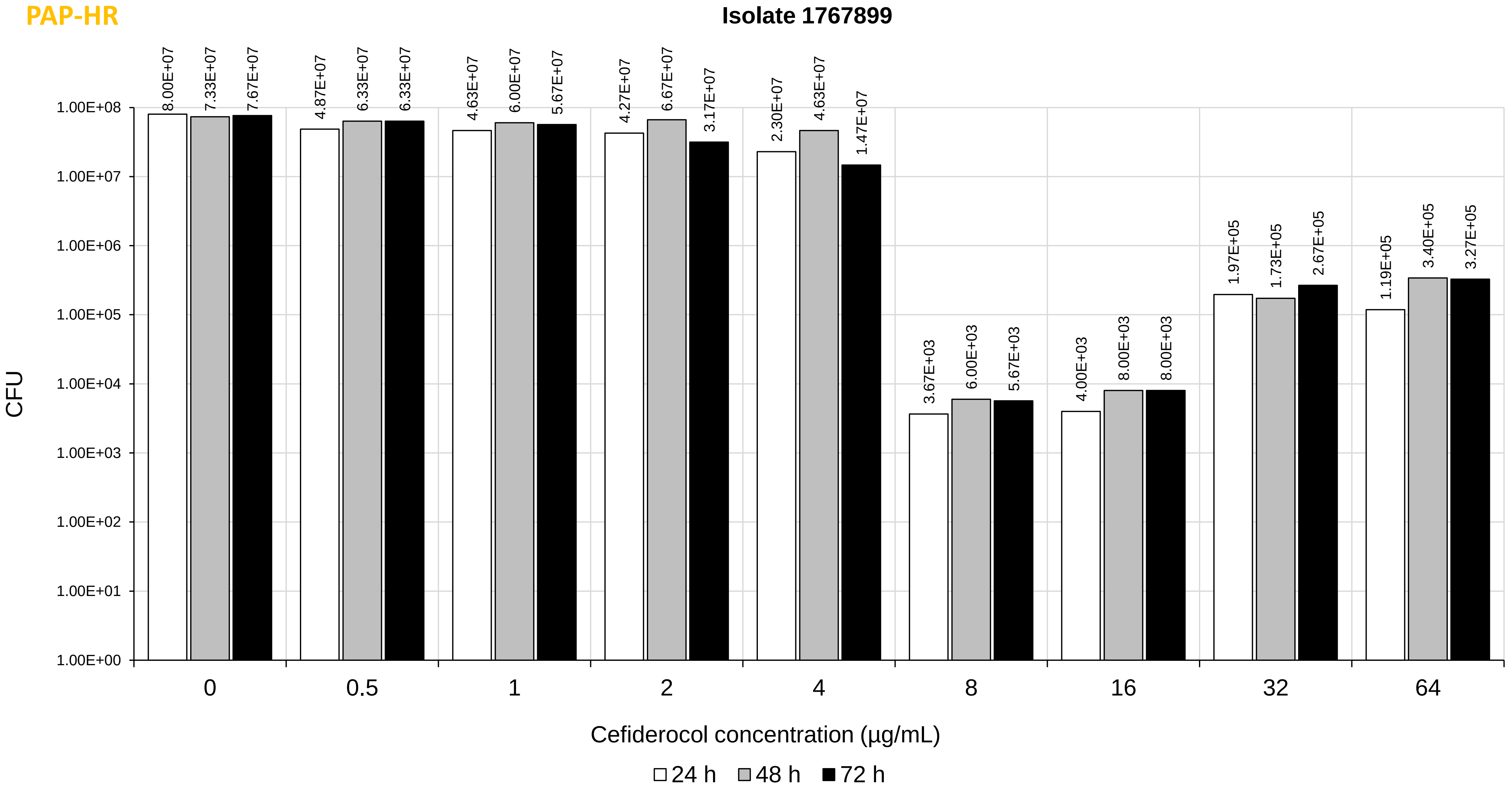

CFU, colony-forming unit; HR, heteroresistant; PAP, population analysis profiling.

Supplementary Fig S1.23.
Growth profile of carbapenem-resistant *Acinetobacter calcoaceticus-baumannii* complex by PAP method

PAP-R

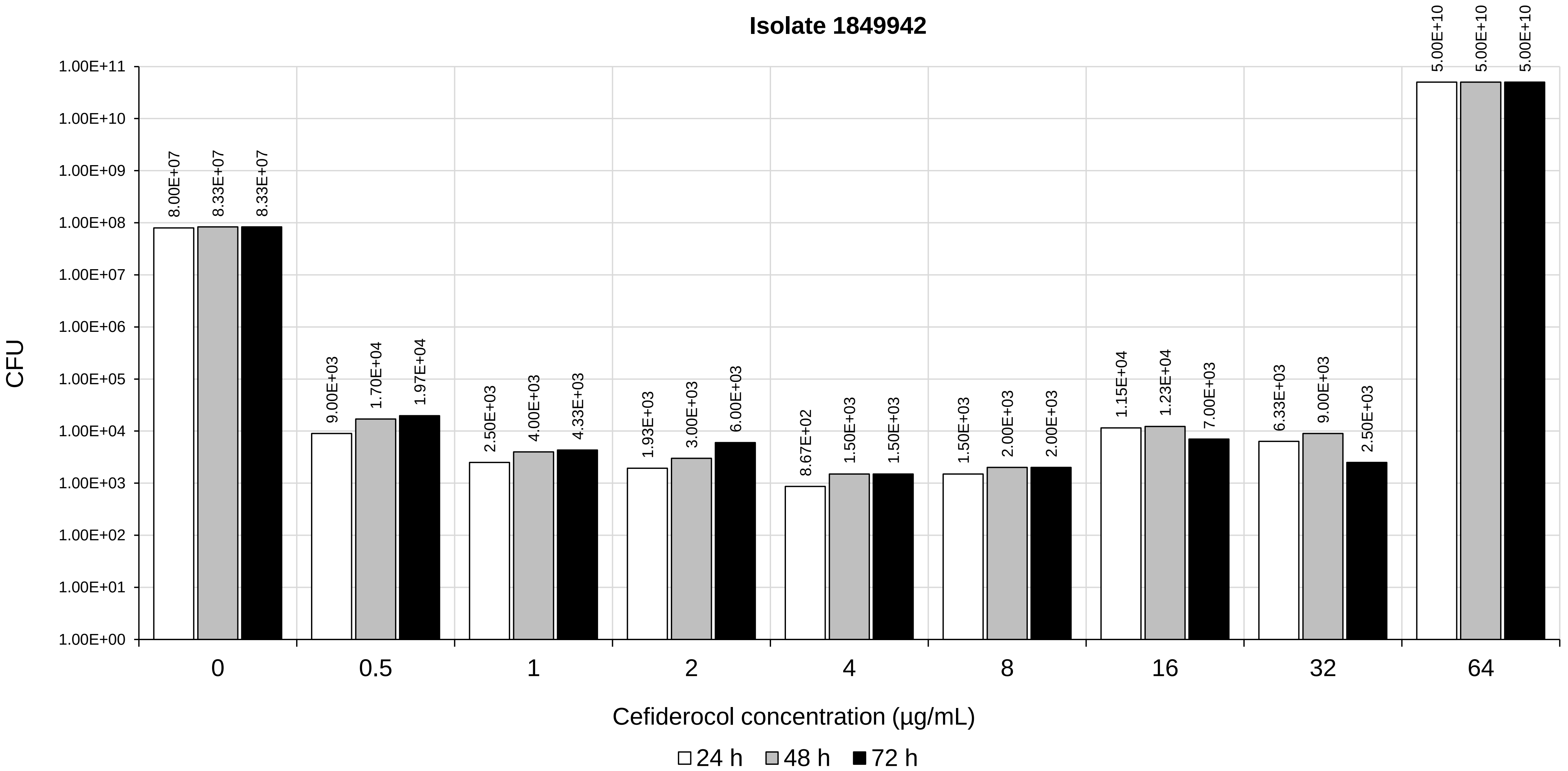

CFU, colony-forming unit; PAP, population analysis profiling; R, resistant.

Supplementary Fig S1.24.
Growth profile of carbapenem-resistant *Acinetobacter calcoaceticus-baumannii* complex by PAP method

PAP-R

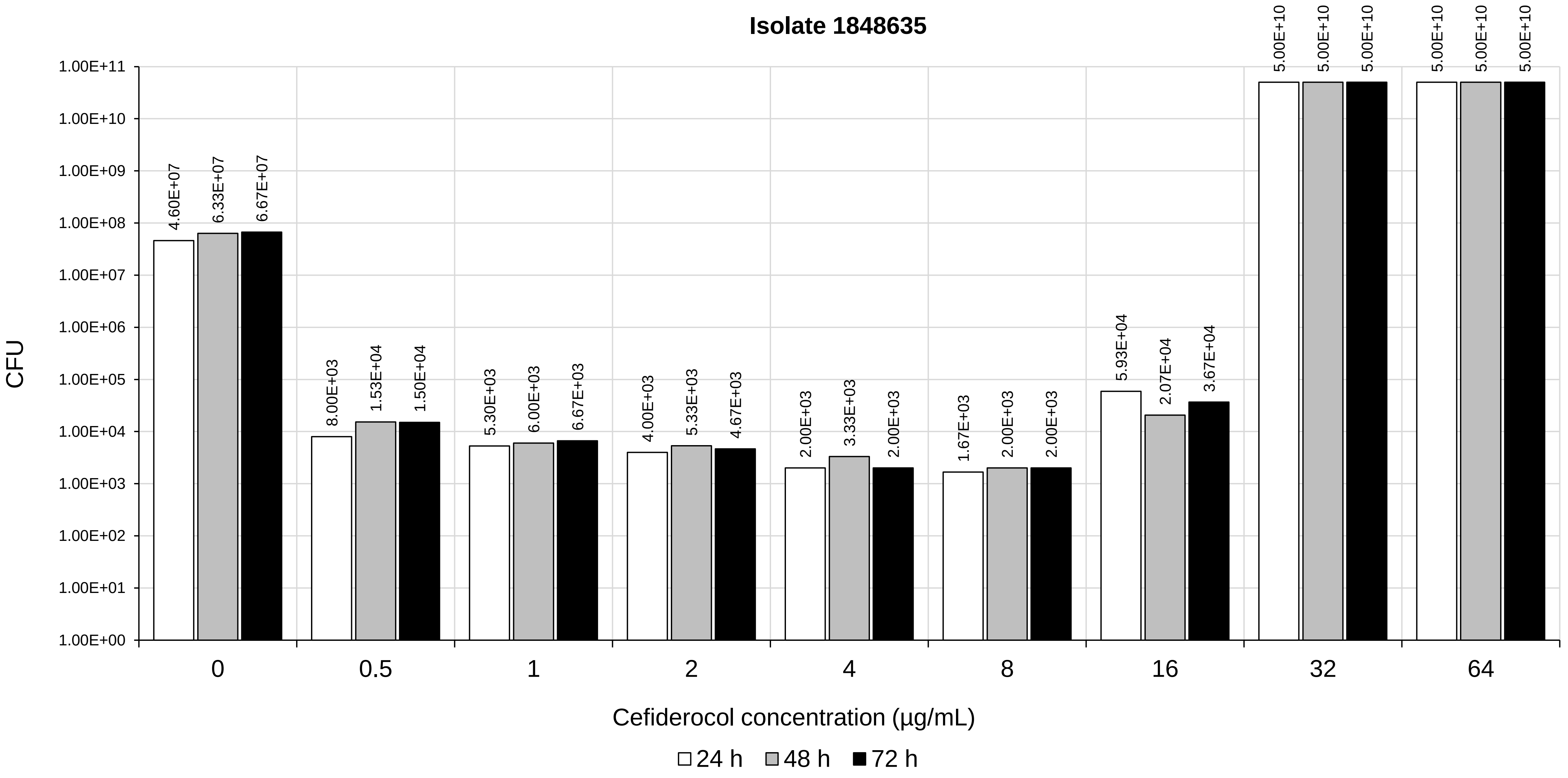

CFU, colony-forming unit; PAP, population analysis profiling; R, resistant.

Supplementary Fig S1.25.
Growth profile of carbapenem-resistant *Acinetobacter calcoaceticus-baumannii* complex by PAP method

PAP-HR

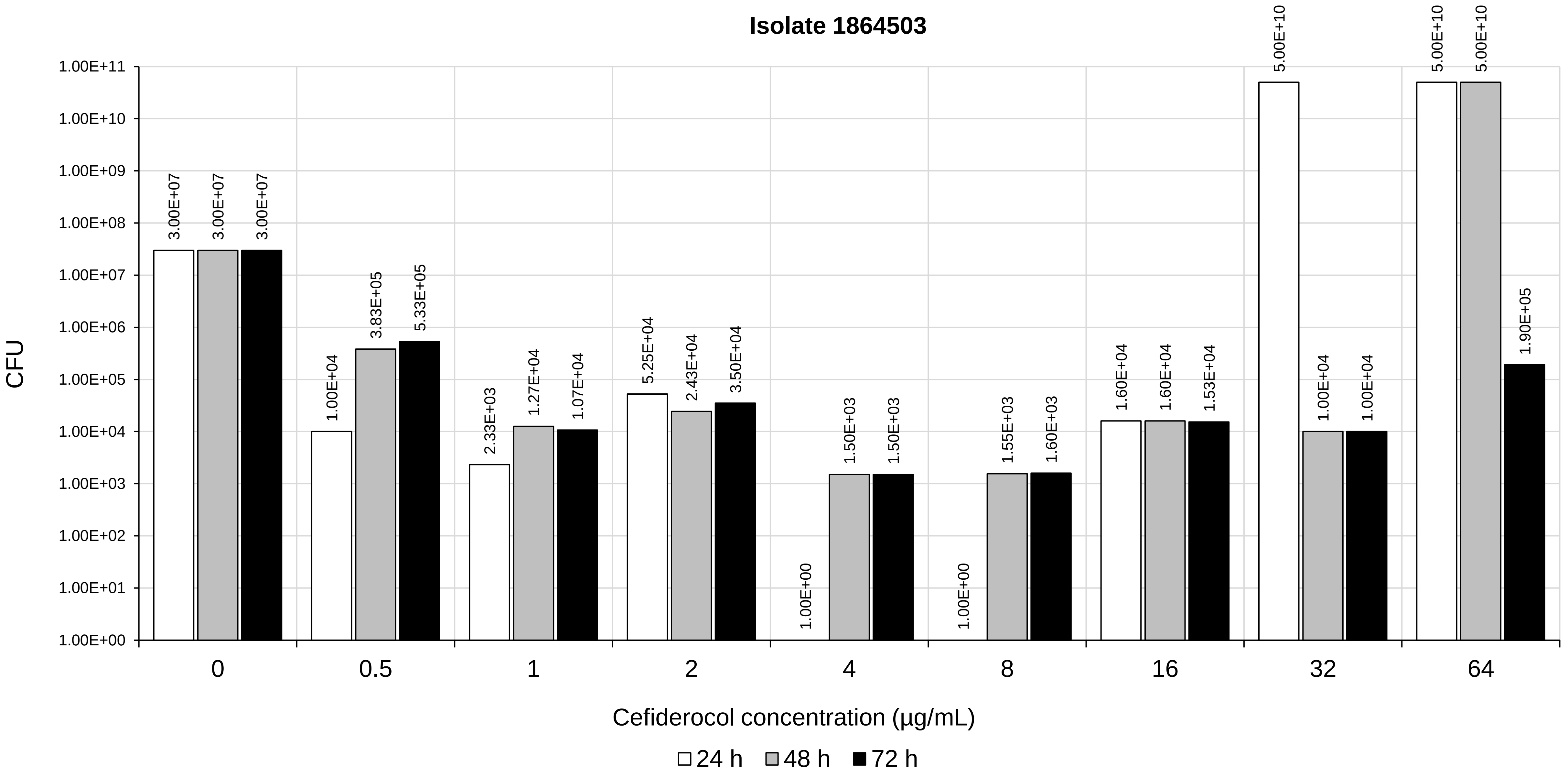

CFU, colony-forming unit; HR, heteroresistant; PAP, population analysis profiling.

Supplementary Fig S1.26.
Growth profile of carbapenem-resistant *Acinetobacter calcoaceticus-baumannii* complex by PAP method

PAP-HR

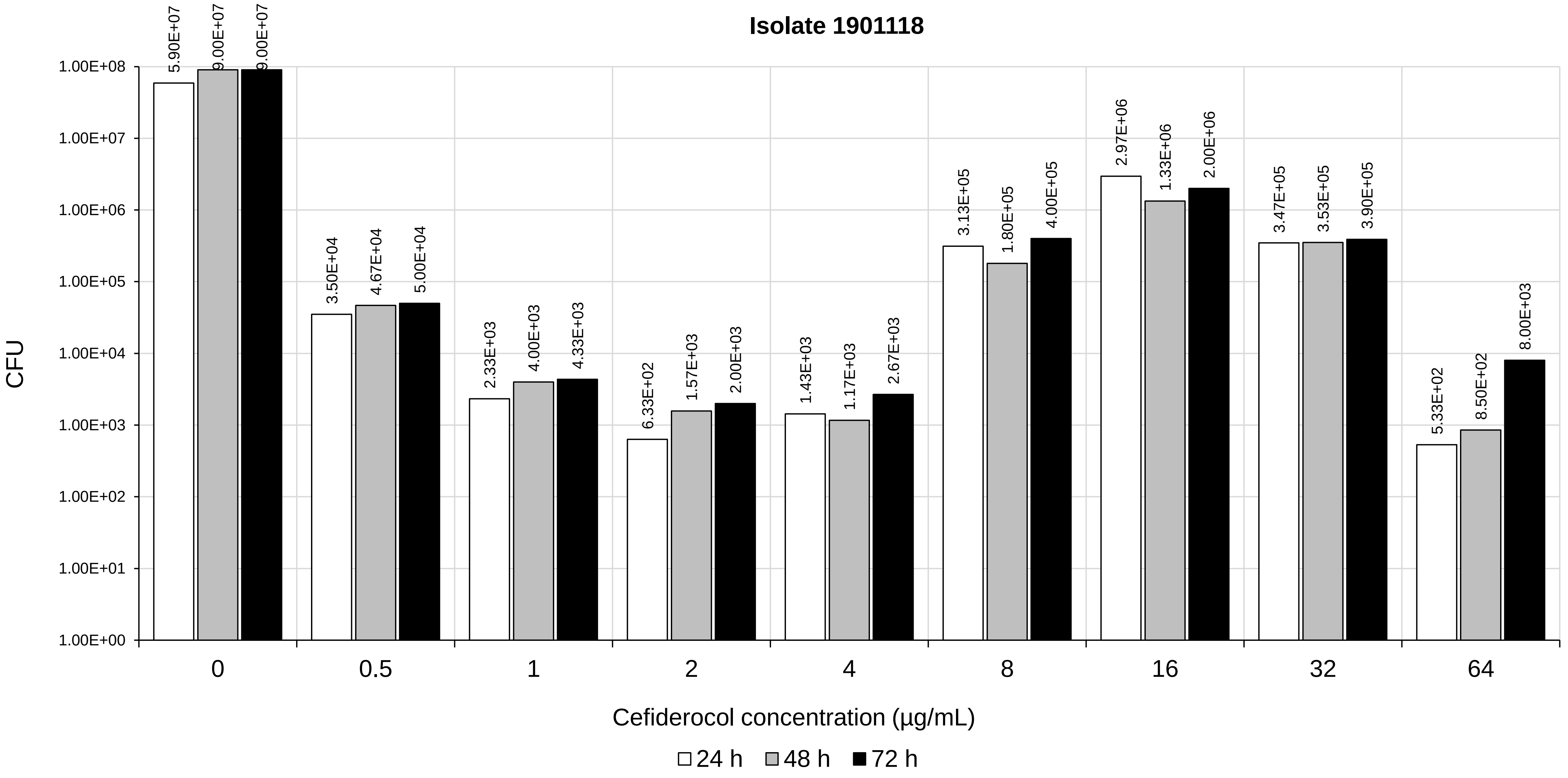

CFU, colony-forming unit; HR, heteroresistant; PAP, population analysis profiling.

Supplementary Fig S1.27.
Growth profile of carbapenem-resistant *Acinetobacter calcoaceticus-baumannii* complex by PAP method

PAP-HR

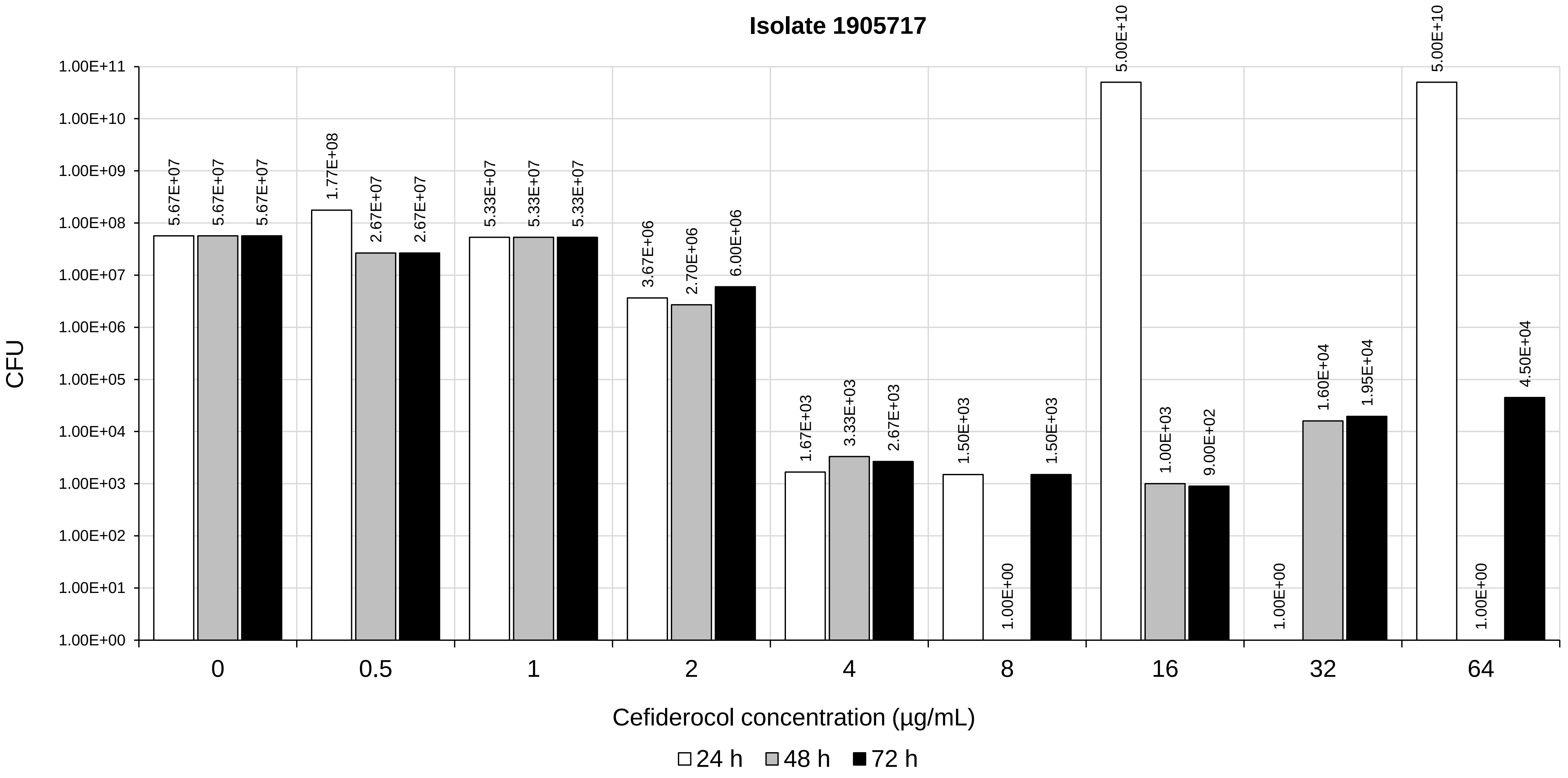

CFU, colony-forming unit; HR, heteroresistant; PAP, population analysis profiling.

Supplementary Fig S1.28.
Growth profile of carbapenem-resistant *Acinetobacter calcoaceticus-baumannii* complex by PAP method

PAP-R

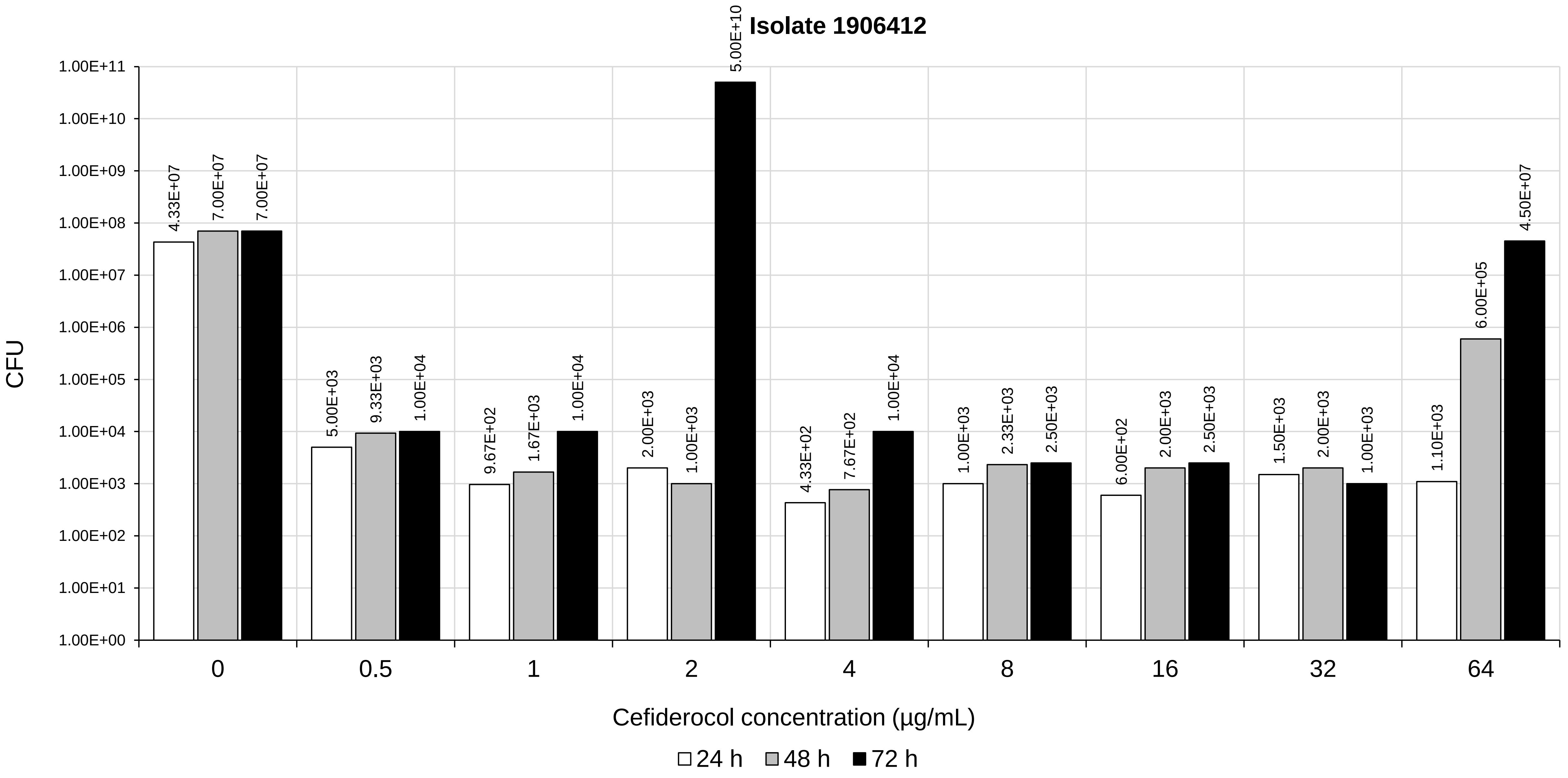

CFU, colony-forming unit; PAP, population analysis profiling; R, resistant.

Supplementary Fig S1.29.
Growth profile of carbapenem-resistant *Acinetobacter calcoaceticus-baumannii* complex by PAP method

PAP-HR

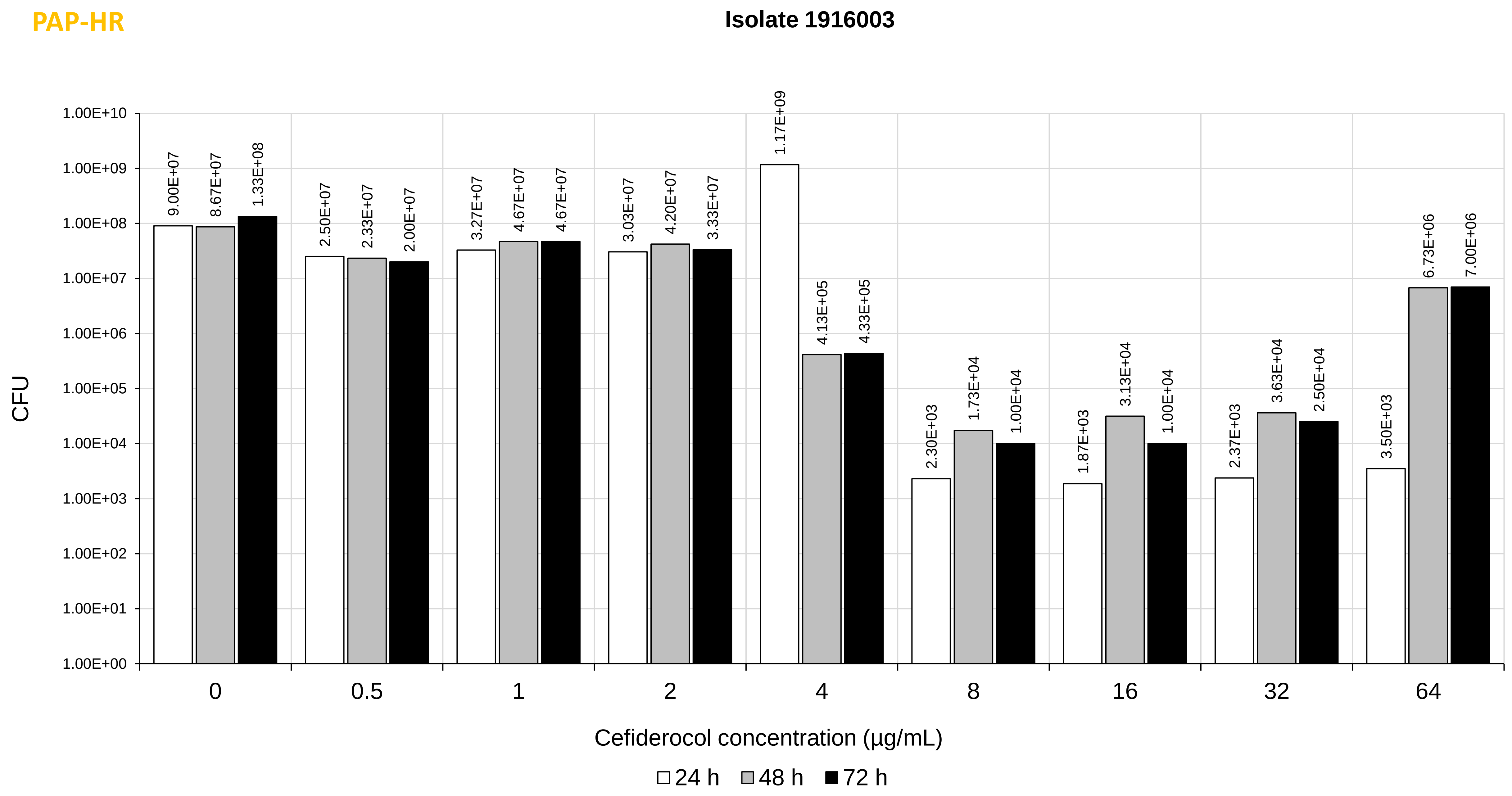

CFU, colony-forming unit; HR, heteroresistant; PAP, population analysis profiling.

Supplementary Fig S1.30.
Growth profile of carbapenem-resistant *Acinetobacter calcoaceticus-baumannii* complex by PAP method

PAP-S

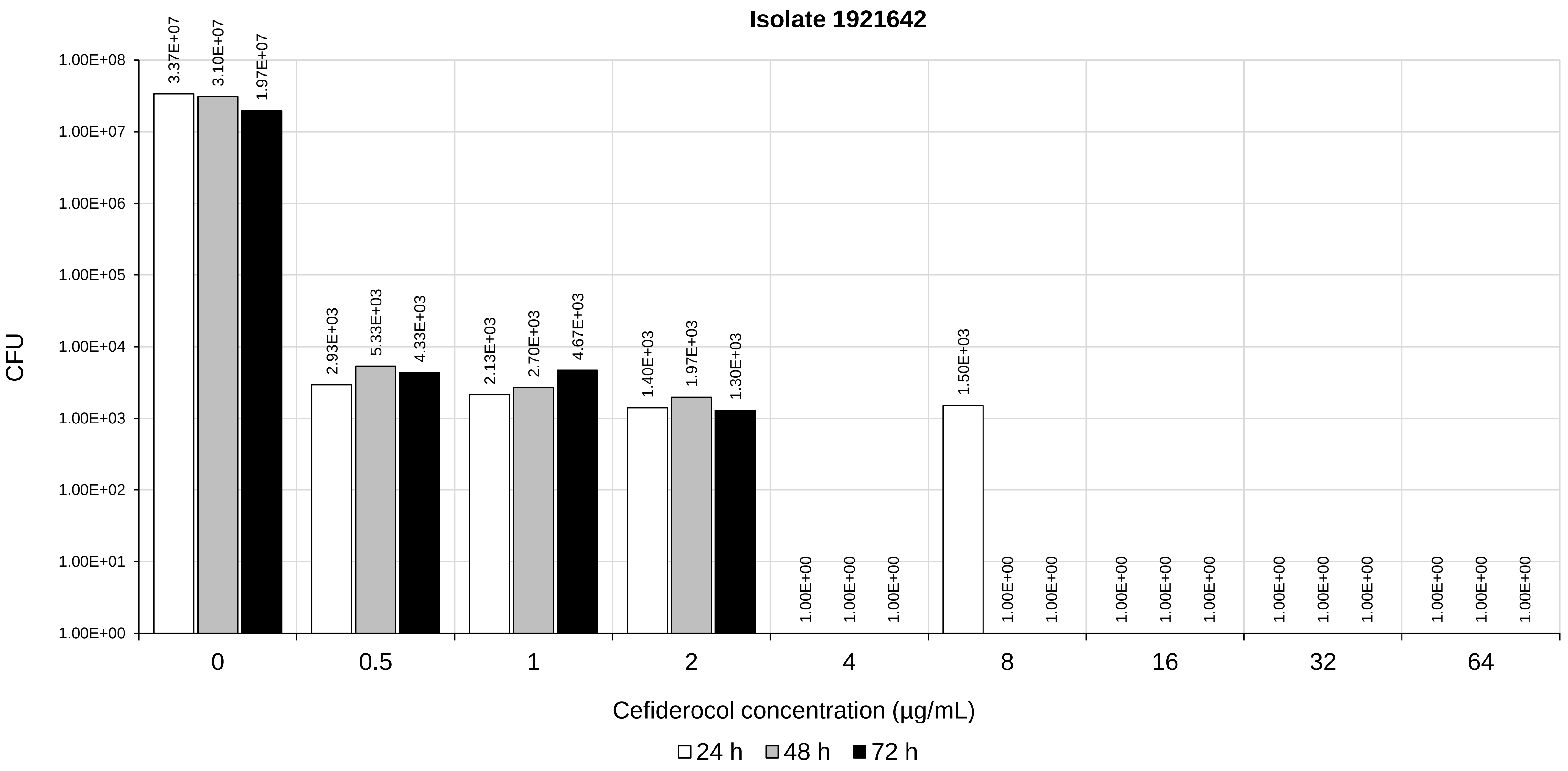

CFU, colony-forming unit; PAP, population analysis profiling; S, susceptible.

Supplementary Fig S1.31.
Growth profile of carbapenem-resistant *Acinetobacter calcoaceticus-baumannii* complex by PAP method

PAP-HR

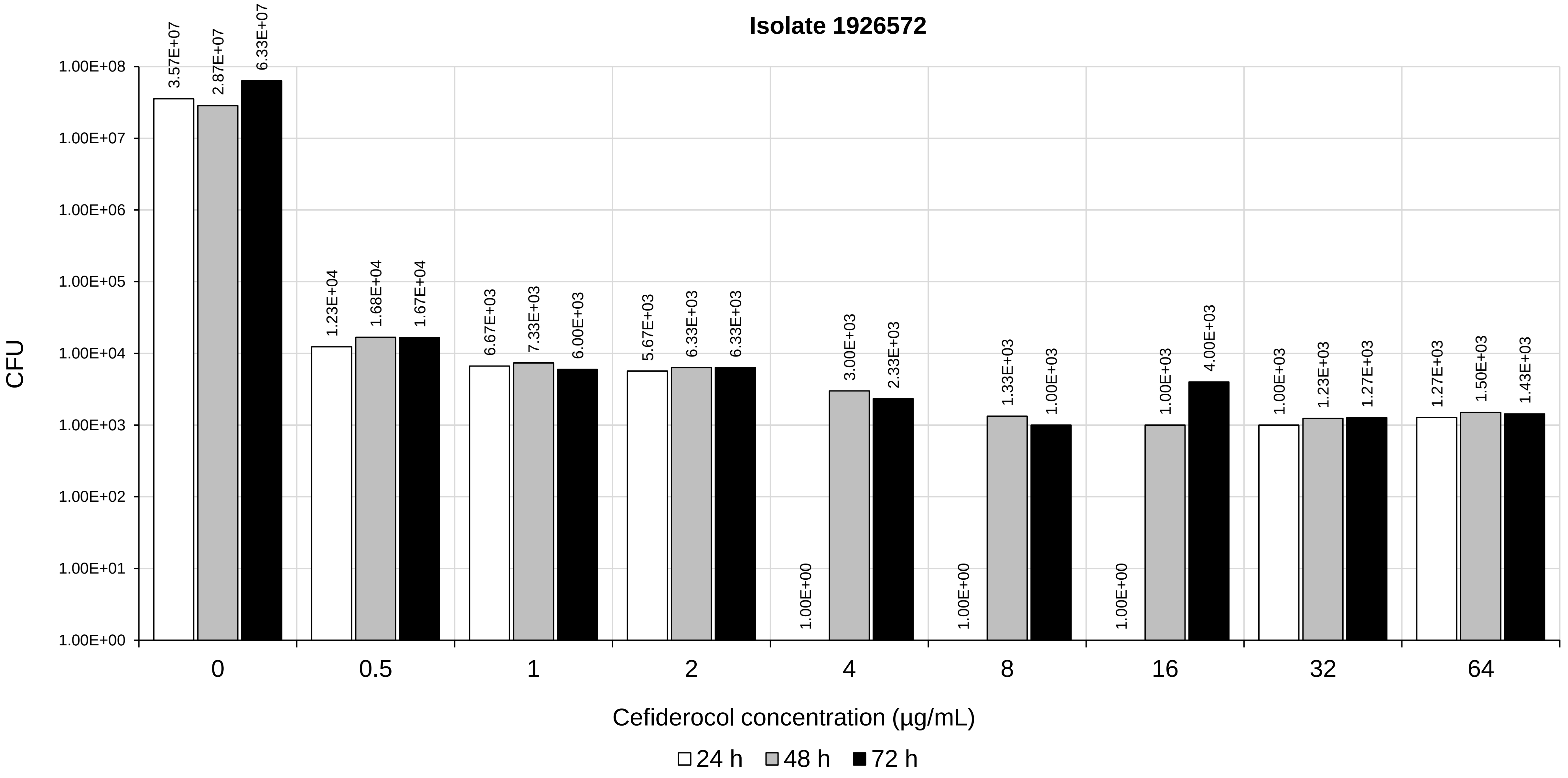

CFU, colony-forming unit; HR, heteroresistant; PAP, population analysis profiling.

Supplementary Fig S1.32.
Growth profile of carbapenem-resistant *Acinetobacter calcoaceticus-baumannii* complex by PAP method

PAP-R

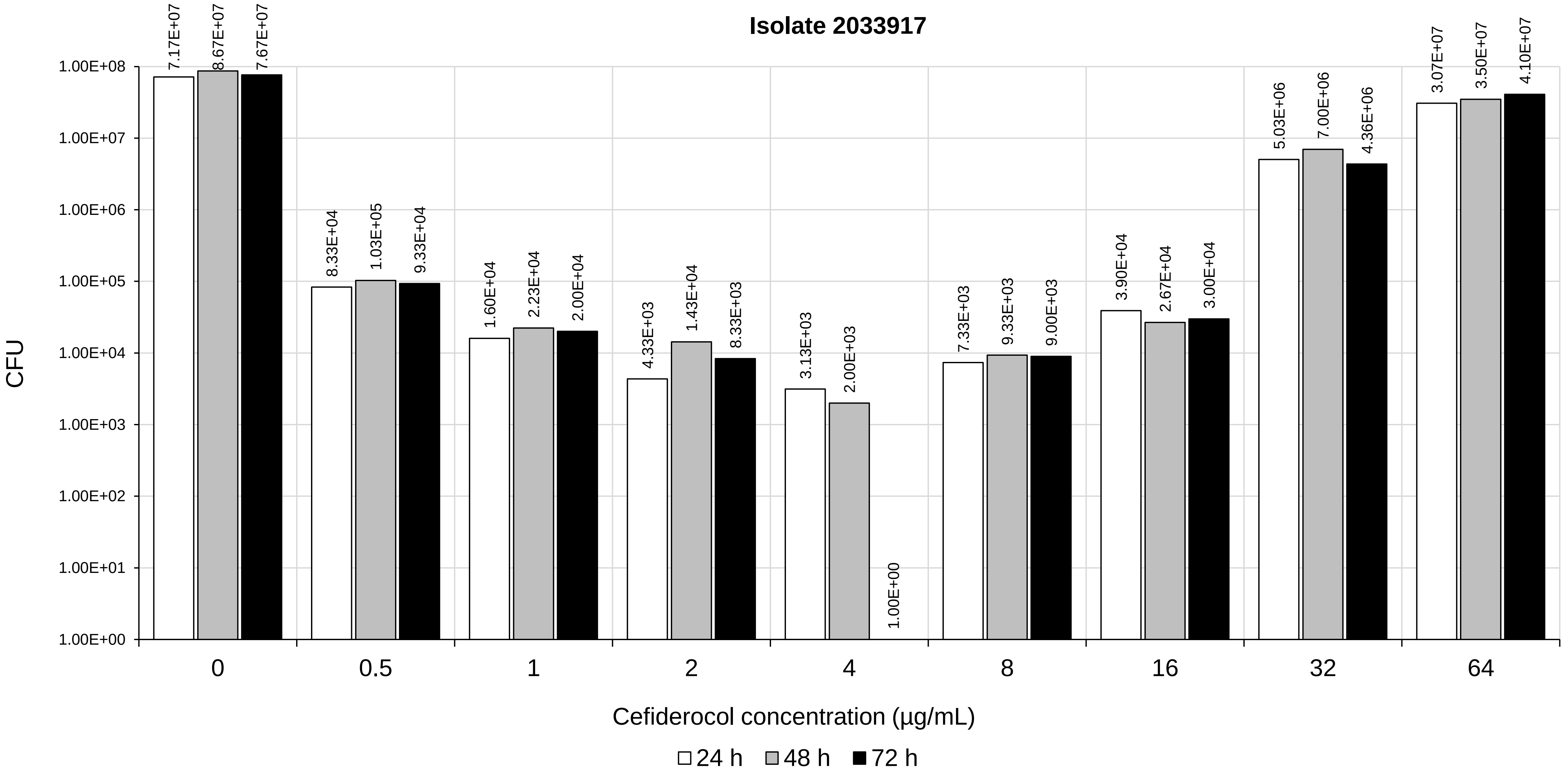

CFU, colony-forming unit; PAP, population analysis profiling; R, resistant.

Supplementary Fig S1.33.
Growth profile of carbapenem-resistant *Acinetobacter calcoaceticus-baumannii* complex by PAP method

PAP-S

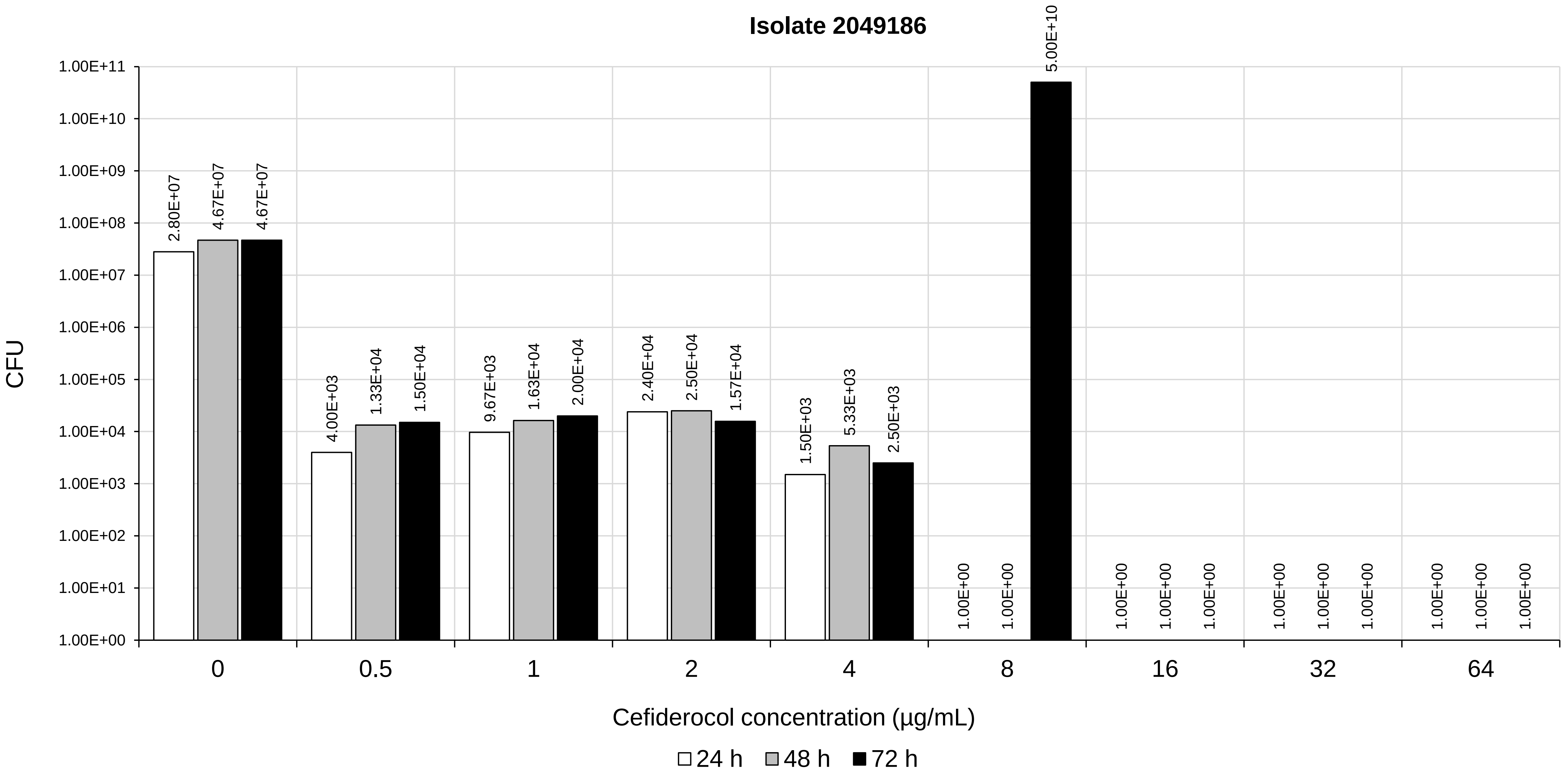

CFU, colony-forming unit; PAP, population analysis profiling; S, susceptible.

Supplementary Fig S1.34.
Growth profile of carbapenem-resistant *Acinetobacter calcoaceticus-baumannii* complex by PAP method

PAP-R

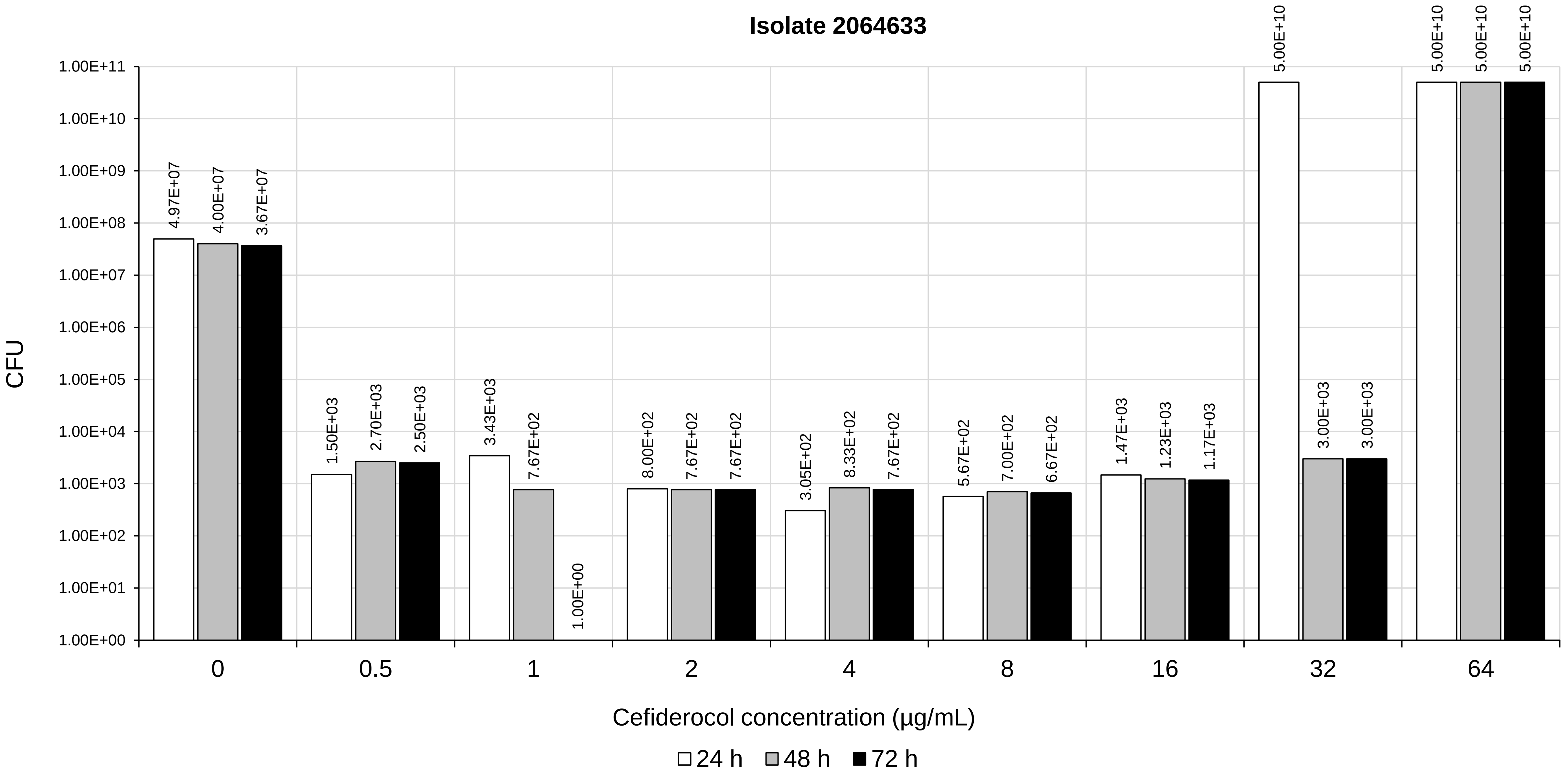

CFU, colony-forming unit; PAP, population analysis profiling; R, resistant.

Supplementary Fig S1.35.
Growth profile of carbapenem-resistant *Acinetobacter calcoaceticus-baumannii* complex by PAP method

PAP-HR

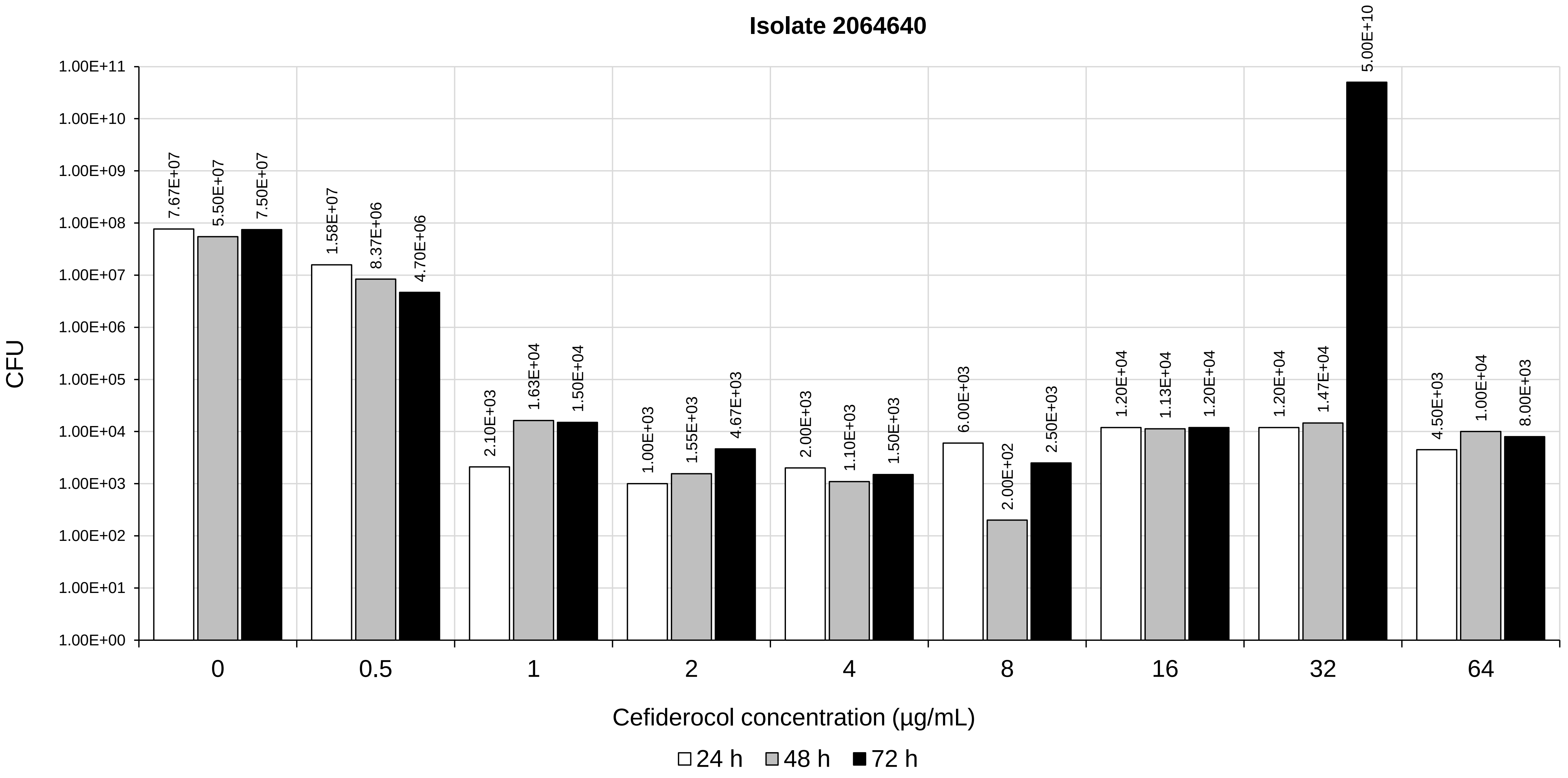

CFU, colony-forming unit; HR, heteroresistant; PAP, population analysis profiling.

Supplementary Fig S1.36.
Growth profile of carbapenem-resistant *Acinetobacter calcoaceticus-baumannii* complex by PAP method

PAP-R

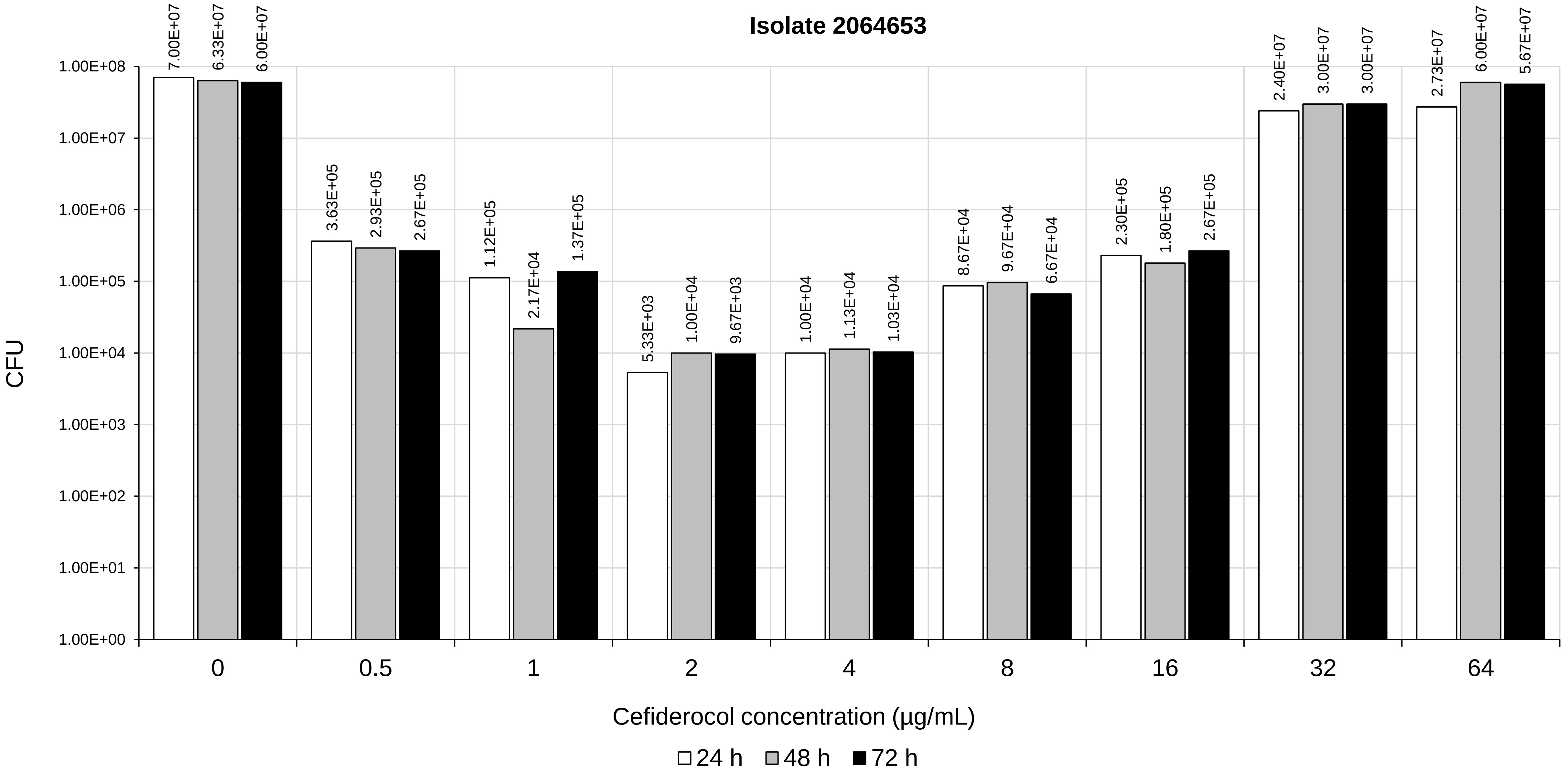

CFU, colony-forming unit; PAP, population analysis profiling; R, resistant.

Supplementary Fig S1.37.
Growth profile of carbapenem-resistant *Acinetobacter calcoaceticus-baumannii* complex by PAP method

PAP-S

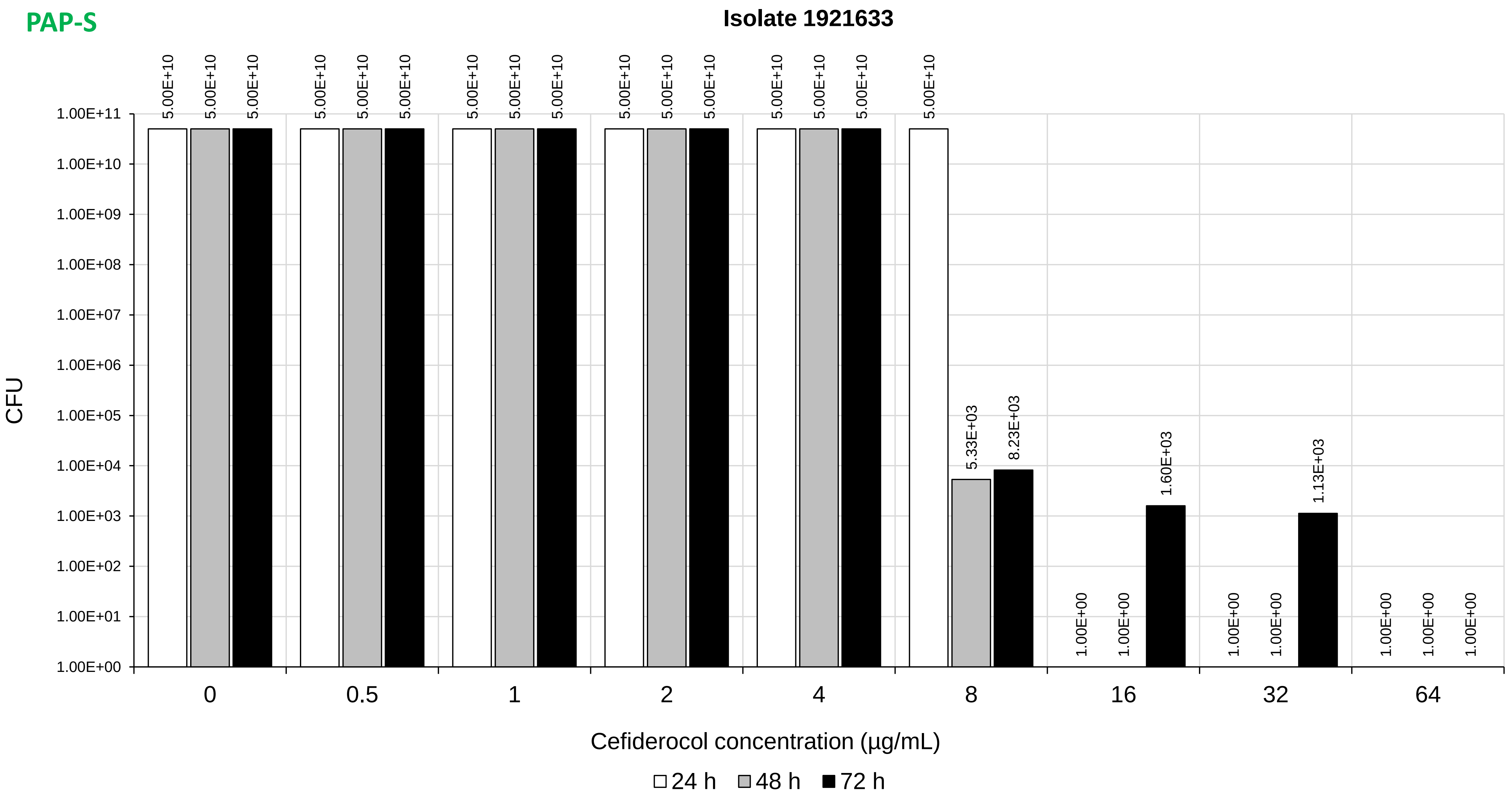

CFU, colony-forming unit; PAP, population analysis profiling; S, susceptible.

Supplementary Fig S1.38.
Growth profile of carbapenem-resistant *Acinetobacter calcoaceticus-baumannii* complex by PAP method
