# Supplementary material for: Heteroresistance to cefiderocol in carbapenem-resistant Acinetobacter baumannii in the CREDIBLE-CR study was not linked to clinical outcomes: a post hoc analysis
Source: Microbiol Spectr. 2023 Nov 15;11(6):e02371-23. doi: 10.1128/spectrum.02371-23 (PMC10714777; doi:10.1128/spectrum.02371-23)
Supplement: Table S1 — Clinical characteristics of patients with carbapenem-resistant Acinetobacter calcoaceticus-baumannii complex who died or survived by end of study in the cefiderocol arm in the CREDIBLE-CR study. [file spectrum.02371-23-s0002.pdf]

**Table S1.** Clinical characteristics of patients with carbapenem-resistant *Acinetobacter calcoaceticus-baumannii* complex who died or survived by end of study in the cefiderocol arm in the CREDIBLE-CR study

| | **Patients with CRAB infection treated with cefiderocol in the CREDIBLE-CR study (N=38)** | |
|---|---|---|
| **Parameters at randomization** | **Patients who died by EOS n=19** | **Patients who survived by EOS n=19** |
| **Age** | | |
| Mean (SD) | 64.0 (17.1) | 54.5 (23.8) |
| Median (range [min–max]) | 69 (24–86) | 67 (23–91) |
| **Sex** | | |
| Male, n (%) | 11 (57.8) | 13 (68.4) |
| Female, n (%) | 8 (42.2) | 6 (31.6) |
| **ICU, n (%)** | 16 (84.2) | 16 (84.2) |
| **Mechanical ventilation, n (%)** | 15 (78.9) | 12 (63.2) |
| **APACHE II** | | |
| Mean (SD) | 18.6 (5.6) | 15.1 (6.0) |
| Median (range [min–max]) | 18 (12–29) | 15 (5–26) |
| **SOFA** | | |
| Mean (SD) | 7.7 (3.9) | 5.3 (3.4) |
| Median (range [min–max]) | 7 (3–17) | 4 (1–12) |
| **CCI** | | |
| Mean (SD) | 7.0 (2.5) | 3.4 (2.3) |
| Median (range [min–max]) | 7 (3–11) | 4 (0–7) |
| **Severe/moderate renal impairment,* n (%)** | 9 (47.4) | 3 (15.8) |
| **Any organ failure,** n (%)** | 15 (78.9) | 7 (36.8) |
| **Ongoing or prior (within 31 days) septic shock** | 8 (42.1) | 1 (5.3) |
| **Sepsis without septic shock** | 2 (10.5) | 0 (0) |

APACHE II, Acute Physiology And Chronic Health Evaluation II; CCI, Charlson Comorbidity Index; CRAB, carbapenem-resistant *Acinetobacter calcoaceticus-baumannii* complex; EOS, end of study; ICU, intensive care unit; SD, standard deviation; SOFA, Sequential Organ Failure Assessment.

*Based on creatinine clearance [1].
**Includes any acute or chronic renal, lung, liver, and/or cardiac failure or insufficiency reported in medical history prior to randomization.

## Reference

1. Bassetti M, Echols R, Matsunaga Y, Ariyasu M, Doi Y, Ferrer R, Lodise TP, Naas T, Niki Y, Paterson DL, Portsmouth S, Torre-Cisneros J, Toyoizumi K, Wunderink RG, Nagata TD. 2021. Efficacy and safety of cefiderocol or best available therapy for the treatment of serious infections caused by carbapenem-resistant Gram-negative bacteria (CREDIBLE-CR): a randomised, open-label, multicentre, pathogen-focused, descriptive, phase 3 trial. Lancet Infect Dis 21:226–240.
